# Trifluoromethoxypyrazines: Preparation and Properties

**DOI:** 10.3390/molecules25092226

**Published:** 2020-05-09

**Authors:** Taras M. Sokolenko, Yurii L. Yagupolskii

**Affiliations:** Institute of Organic Chemistry, NAS of Ukraine, Murmans’ka St. 5, Kyiv 02660, Ukraine; taras_sk@ukr.net

**Keywords:** pyrazine, trifluoromethoxy group, chlorination, fluorination, antimony trifluoride, coupling reactions

## Abstract

The incorporation of the trifluoromethoxy group into organic molecules has become very popular due to the unique properties of the named substituent that has a “pseudohalogen” character, while the chemical properties of the synthesized compound, especially heterocycles with such a group, are less studied. As trifluoromethoxy-substituted pyrazines are still unknown, we have developed efficient and scalable methods for 2-chloro-5-trifluoromethoxypyrazine synthesis, showing the synthetic utility of this molecule for Buchwald-Hartwig amination and the Kumada-Corriu and Suzuki and Sonogashira coupling reactions. Some comparisons of chlorine atom and trifluoromethoxy group stability in these transformations have been carried out.

## 1. Introduction

Among fluorine-containing substituents, the trifluoromethoxy group have gained considerable interest from the modern synthetic audience due to its high influence on molecules for biological and material sciences [1,2]. This moiety is often viewed as a privileged substituent in the field of medicinal chemistry and is routinely considered during drug design [3]. This growing interest in trifluoromethyl ethers is caused by the very peculiar characteristics of the CF_3_O group. This substituent possesses electron-withdrawing properties by the induction effect and an electron-donating one by the mesomeric for chlorine atoms. For this reason, it has been named “super-halogen” or “pseudo-halogen” [2,4,5]. Moreover, it is one of the most hydrophobic substituents (π(OCF_3_) = +1.04 compared to π(OCH_3_) = −0.02) [6]. Additionally, the unique conformation of trifluoromethoxyarenes, in which the OCF_3_ moiety is orthogonal to the ring plane, must be noted [7,8].

The trifluoromethoxy group attached to aromatics and heteroaromatics is relatively inert and demonstrates the highest stability towards heating, acidic or basic conditions among other fluorine-containing substituents, such as SCF_3_, CF_3_, CHF_2_, OCHF_2_, SHCF_2_, and OCH_2_F [9,10,11,12]. The synthetic utility of pyridine with the OCF_3_ group was well documented by Leroux and coworkers [8]. It was shown that the trifluoromethoxy group in the 3-rd and even 2-nd position of the ring is tolerant to various reaction conditions, including metallation and catalytic reduction. The authors showed that the trifluoromethoxy group in the 4-th position is less stable and undergoes replacement by bromine atoms under the action of TMSBr. The trifluoromethoxy group easily undergoes nucleophilic displacement when attached to a electron-deficient benzene ring, which was demonstrated by Langlois and coworkers for the example of dinitro-trifluoromethoxybenzene [5].

Taking into account that pyrazine derivatives play an important biological role as food components and scent markers [13,14,15], as well as a number of pharmaceuticals (antitubarcular pyrazinamide, anti-HIV oltipraz, proteasome inhibitor bortezomib, narcotic addiction varenceline, fungal antibiotic aspergillic acid, pulmonary heart disease drug ligustrazine, hypnotic eszopiclone, and eye drops for glaucoma or ocular hypertension brimonidine [13,16]), the trifluoromethoxylated pyrazines family provide perspective molecules for investigation.

Since the first publication in 1955 [17], the main classical methods of trifluoromethoxy ether syntheses are based on chlorination/fluorination techniques or the oxidative desulfofluorination of xanthates [8,18,19,20,21,22]. The range of novel approaches, including the electrophilic trifluoromethylation of oxygen atoms [3,23,24,25,26,27,28] or the incorporation of the trifluoromethoxy group [5,29,30,31,32,33,34], were developed during recent years and successfully applied for aliphatic, aromatic, and heteroaromatic substrates. Nevertheless, pyrazines with such a group are still unknown.

A pyrazine ring is more electron-deficient than a pyridine one due to the influence of the second nitrogen atom [13]. The highly electron-deficient nature of the pyrazine system allows the successful use of the aromatic nucleophilic substitution and cross-coupling reactions for the functionalization of halogenated pyrazines [13,16,35]. 2-Chloro-5-trifluoromethoxypyrazine is a unique compound because both substituents—chlorine atoms and the trifluoromethoxy group—are in equivalent positions. This specific feature opens the possibility to compare the mentioned moieties in various types of reactions and raises the question: are the pyrazines bearing trifluoromethoxy group stable and suitable for synthetic transformations?

## 2. Results and Discussion

**Scheme 1:**
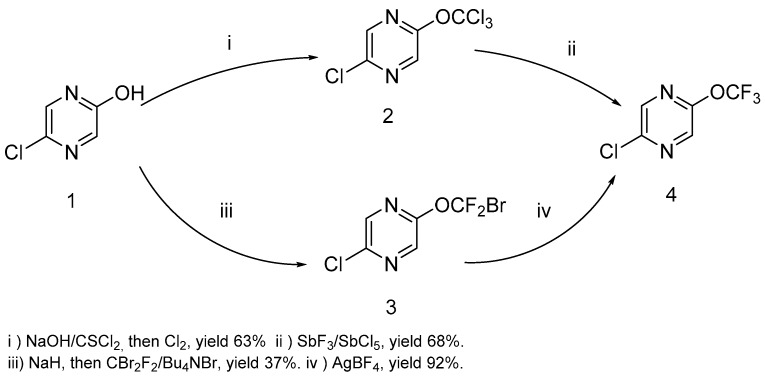
Preparation of the 2-chloro-5-trifluoromethoxypyrazine.

We have prepared 2-chloro-5-trifluoromethoxypyrazine **4** by the action of thiophosgene on hydroxypyrazine, with further chlorination and a chlorine/fluorine exchange using antimony trifluoride (Scheme 1). We used an alternative route based on the halophilic alkylation of pyrazine by dibromodifluoromethane, followed by a bromine/fluorine exchange with silver tetrafluoroborate (Scheme 1). The last method is less favorable due to the low yield in the first stage and the expensive reagents used. Nonetheless, both methods are suitable for a large scale experiment (pyrazine **4** was prepared in 20–25 g scale) in common laboratory glass equipment.

It must be noted that the presence of chlorine atoms in the pyrazine ring is critical for the success of both methods. We have found that trichloromethoxy and bromodifluoromethoxypyrazine cannot be prepared by these methods starting from 2-hydroxypyrazine. The same peculiarity was shown by Leroux and coworkers for a trifluoromethoxypyridine preparation [8].

**Scheme 2:**
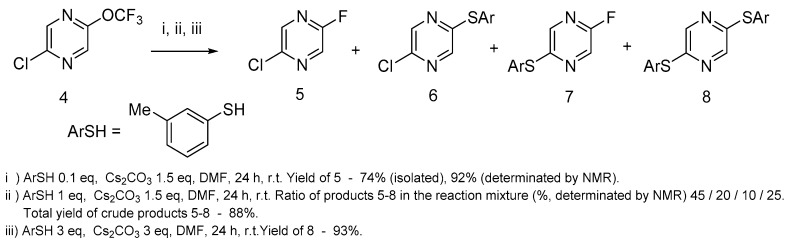
Reaction of 2-chloro-5-trifluoromethoxypyrazine with 3-methylthiophenol.

We have studied the 2-chloro-5-trifluoromethoxypyrazine behavior with nucleophiles using 3-methylthiophenol as an example of *S-*nucleophile. If one equivalent of 3-methylthiophenol was used in this reaction, 2-chloro-5-fluoropyrazine **5** was the main fluorinated product (Scheme 2), but a range of products with thiophenol moiety were also formed in these conditions. Surprisingly, 2-chloro-5-fluoropyrazine **5** was the main product when pyrazine **4** reacted with 10 mol% 3-methylthiophenol and an excess of cesium carbonate at room temperature. In the absence of thiophenol, this reaction did not occur even after 1 week of stirring at r.t. Pyrazine **8** was the single product when this reaction was performed with an excess of 3-methylthiophenol.

**Scheme 3:**
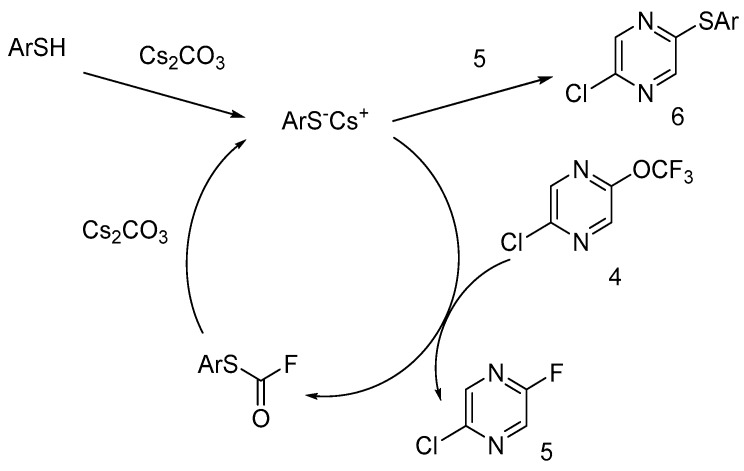
Probable mechanism of OCF_3_ group destruction with the S-nucleophiles.

Taking into account the destruction of OCF_3_ moiety in the presence of catalytic amounts of thiophenolate anions, we proposed the following scheme for trifluorometoxy group degradation (Scheme 3): the thiophenolate anion reacts with trifluorometoxy pyrazine **4,** with an S-aryl fluorocarbamate formation that can be recovered to thiophenolate by the action of cesium carbonate. The nucleophilic substitution of the fluorine atom of pyrazins **5** and **7** or the chlorine atom of pyrazines **5** and **6** is the route of removing thiophenol from the catalytic cycle (only pyrazine **5** was shown in Scheme 3 as an example). A high yield of fluoropyrazine **5** is evidence of the high catalytic activity of thiophenol for this reaction.

**Scheme 4:**
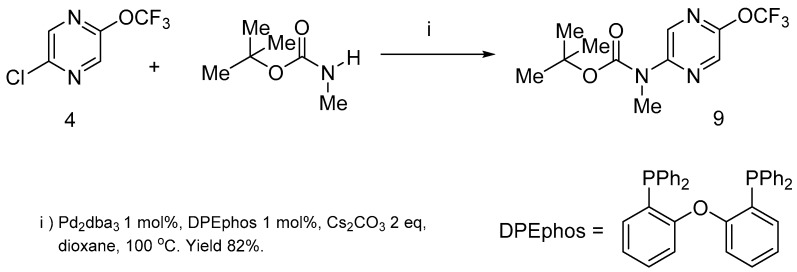
2-Chloro-5-trifluoromethoxypyrazine **4** in the Buchwald-Hartwig amination reaction.

The nucleophilic substitution of chlorine atoms in pyrazine **4** by diethylamine as an example of *N-* nucleophiles did not occur at room temperature. The prolonged heating (24 h) of the reaction mixture in various solvents at 100 °C led to the complete destruction of the starting material. Nevertheless, the chlorine atom of this molecule was successfully replaced by nitrogen nucleophile under Buchwald-Hartwig amination reaction conditions [35,36,37]. Palladium dibenzoylacetonate (Pd_2_dba_3_)/DPEphos catalysis took place and the Boc-protected amine **9** was prepared in high yield (Scheme 4).

**Scheme 5:**
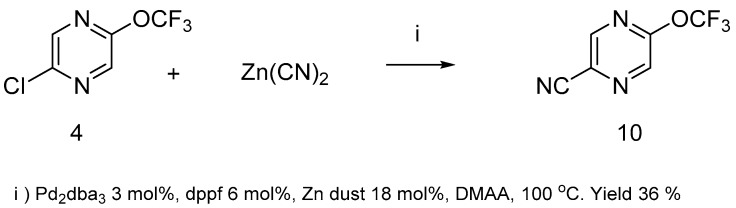
2-Chloro-5-trifluoromethoxypyrazine **4** in the palladium-catalyzed cyanation reaction.

The replacement of chlorine atom in compound **4** with the nitrile group using potassium cyanide failed, however pirazinenitrile **10** was prepared with zinc cyanide under Pd_2_dba_3_/diphenylphosphinoferrocene (*dppf*) catalysis (Scheme 5).

Alkylpyrazines, especially bearing methoxy group ones, are compounds of great interest due to their odor properties [38,39,40]. For instance, the odor threshold of 2-methoxy-3-*sec*-buthyl-pyrazine in water solution is 1 part per 10^12^ parts of water [39].

**Scheme 6:**
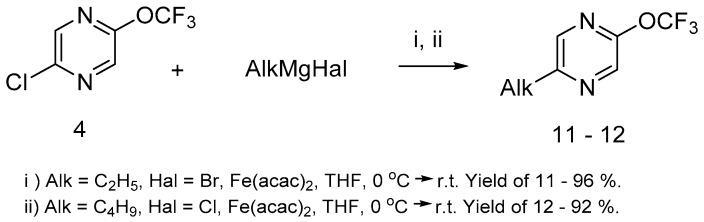
2-Chloro-5-trifluoromethoxypyrazine **4** in the iron-catalyzed Kumada–Corriu coupling.

We have shown that the alkyl pirazines-bearing trifluoromethoxy group can be prepared in high yield by Kumada–Corriu coupling [41,42,43] with iron acetylacetonate (Fe(acac)_2_) as a catalyst (Scheme 6). It must be noted that both alkylmagnesium halides (bromide or chloride) are suitable for this reaction.

**Scheme 7:**
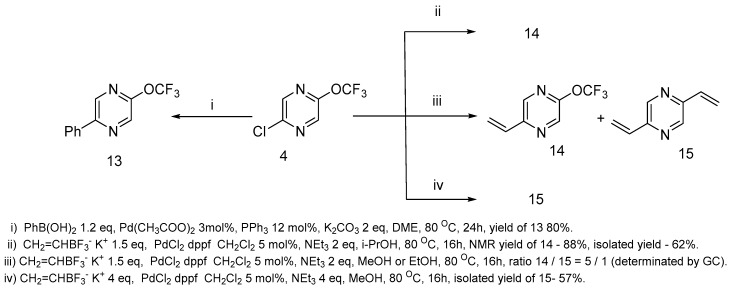
2-Chloro-5-trifluoromethoxypyrazine **4** in Suzuki coupling.

Suzuki coupling [44] is a powerful method for pyrazine derivatization [35]. We have found that 2-chloro-5-trifluoromethoxypyrazine **4** is a suitable substrate for coupling with boronic acids as well as with trifluoroborates. The reaction with phenylboronic acid resulted in phenylpyrazine **13** formation in a high yield (Scheme 7). In the case of potassium ethenyl trifluoroborate, the result of the reaction is dependent on the solvents used (Scheme 7). We tested various solvents for this reaction (DMF, MeOH, EtOH, *i-*PrOH). In DMF, unidentified products were formed. Meanwhile, in the cases of MeOH and EtOH, the reaction resulted in the substitution of the chlorine atom to give product **14**, as well as both the chlorine atom and trifluoromethoxy group removal with the pyrazine **15** formation. Bis-ethenyl pyrazine **15** was formed selectively if a large excess of potassium ethenyl trifluoroborate was used. Monoethenyl pyrazine **14** was produced selectively when *i-*PrOH was used as the solvent. Unfortunately, it is difficult to isolate the product in a pure state in this case, and pyrazine **14** was obtained only with a 62% yield.

**Scheme 8:**
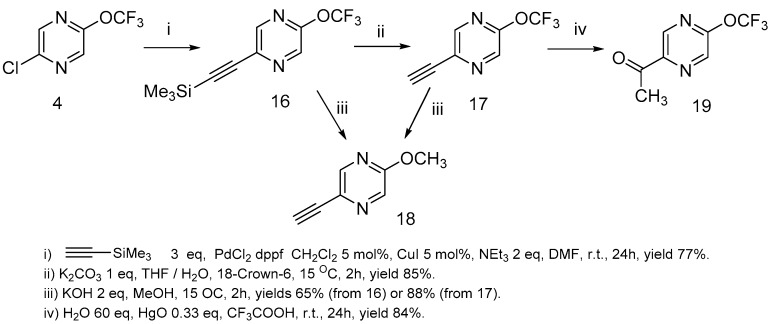
2-Chloro-5-trifluoromethoxypyrazine Sonogashira coupling and Kucherov hydration.

The reaction of 2-chloro-5-trifluoromethoxypyrazine **4** with ethynyltrimethylsilane under Sonogashira [45] reaction conditions led to 2-ethynyl-5-trifluoromethoxypyrazine **16** formation in a high yield (Scheme 8).

Ethynylpyrazine **16** can be successfully desililated by the action of potassium carbonate under heterogeneous reaction conditions in the THF/water mixture. When the desililation of pyrazine **16** was performed with KOH in a methanol/water solution, as was described in the literature [46], a mixture of trifluoromethoxypirazine **17** and methoxypyrazine **18** was obtained. Methoxypyrazine **18** was prepared starting from trifluoromethoxypyrazine **17** or sililated pyrazine **16** by the action of KOH in methanol. The hydration of acetylene **17** via the Kucherov reaction [47,48] resulted in 2-acethyl-5-trifluoromethoxy-pyrazine **19** formation in a high yield (Scheme 8).

## 3. Materials and Methods

^1^H-NMR spectra were recorded at 300 MHz with a Varian VXR-300 spectrometer (Varian Inc., Palo Alto, CA, USA), at 500 MHz with a Bruker AVANCE DRX 500 instrument (Bruker, Billerica, MA, USA), or at 400 MHz with a Varian UNITY-Plus 400 spectrometer (Varian Inc., Palo Alto, CA, USA).^13^C-NMR-spectra (proton decoupled) were recorded on a Bruker AVANCE DRX 500 instrument at 125 MHz, or at 100 MHz with a Varian UNITY-Plus 400 spectrometer (Varian Inc., Palo Alto, CA, USA). ^19^F-NMR spectra were recorded at 376 MHz with a Varian UNITY-Plus 400 spectrometer (Varian Inc., Palo Alto, CA, USA). The chemical shifts are given in ppm relative to Me_4_Si and CCl_3_F, respectively, as internal or external standards. The ^1^H-, ^13^C-, and ^19^F-NMR spectra of the compounds can be found in the Supplementary Materials. The LC-MS spectra were registered on an “Agilent 1100 Series” instrument with a diode-matrix and mass-selective detector “Agilent 1100 LS/MSD SL” (ionization method: chemical ionization at atmospheric pressure; ionization chamber operation conditions: simultaneous scanning of positive and negative ions in the range 80–1000 *m*/*z*, Agilent Technologies, Santa Clara, CA, USA). The GC-MS spectra were registered on a Hewlett-Packard HP GC/MS 5890/5972 instrument (EI 70 eV) (Philips, Bothell, WA, USA). The melting points were determined in open capillaries using an SMP3 instrument (Stuart Scientific Bibby Sterlin Ltd., Stone, Staffordshire, UK). An elemental analysis was performed in the Analytical Laboratory of the Institute of Organic Chemistry, NAS, Ukraine, Kyiv.

Unless otherwise stated, commercially-available reagents were purchased from Enamine Ltd. (Kyiv, Ukraine) and were used without purification. The solvents were purified according to the standard procedures [49]. Antimony trifluoride was sublimated immediately prior to use. The 2-Hydroxy-5-chloropyrazine **1** was prepared starting from 2-amino-5-chloropyrazine according to method [50] in a 37 g scale.

All the reactions were performed in an argon atmosphere. For the column chromatography, Merck Kieselgel 60 silica gel (Merck, Darmstadt, Germany) was used. Thin-layer chromatography (TLC) was carried out on aluminum-backed plates coated with silica gel (Merck Kieselgel 60 F254, Merck, Darmstadt, Germany).

### 3.1. 2-Chloro-5-(trichloromethoxy)pyrazine ***2***

5-Chloropyrazin-2-ol **1** (40 g, 0.31 mol) was added in portions to the solution of NaOH (14.1 g, 0.35 mol) in water (250 mL) at 5–10 °C. The mixture was cooled to 0 °C and the solution of thiophosgene (35.3 g, 0.31 mol) in chloroform (250 mL) was added dropwise over 1 h under vigorous stirring. After the addition was complete, the mixture was vigorously stirred for a further 3 h at 0 °C before being extracted with chloroform (5 × 100 mL). The combined organic layers were washed with water and dried with MgSO_4_. After the dehydration agent had been filtered off, the mixture was saturated with chlorine and stirred for 3 days at 25 °C. The excess of chlorine was removed with nitrogen gas stream, the solvent was distilled off in a vacuum (300 mbar), and the residue was distilled in a vacuum, yielding trichloromethoxypyrazine **2** as a colorless oil or solid. The yield was 47.9 g (63%); b.p. 100–101 °C (1 mbar); m.p. 26–28 °C. ^1^H-NMR (400 MHz, CDCl_3_) δ 8.32 (s, 1H, H_Py_), 8.37 (s, 1H, H_Py_). ^13^C-NMR (126 MHz, CDCl_3_) δ 108.9 (s, OCCl_3_), 136.0, 140.8, 145.6, 154.0. GC-MS, 70 eV, *m*/*z* (rel. int.): 248 (100) [M(3^35^Cl + ^37^Cl)]^+^, 246 (80) [M(4^35^Cl)]^+^, 250 (50) [M(2^35^Cl + 2^37^Cl)]^+^. Anal. Calcd. for C_4_H_2_ClFN_2_: Cl, 57.20; Found: Cl, 57.26.

### 3.2. 2-(Bromodifluoromethoxy)-5-chloropyrazine ***3***

To the solution of 5-chloropyrazin-2-ol (40 g, 0.31 mol) and tetrabutylammonium bromide (4.9 g, 15 mmol) in anhydrous DMF (300 mL), NaH (60% in mineral oil) (14.7 g, 0.37 mol) was added in portions at 0 °C and the mixture was stirred for 2 h at r.t. The mixture was cooled to −30 °C and dibromodifluoromethane (257 g, 1.23 mol) was added dropwise. The mixture was carefully warmed and stirred at 30–35 °C for 6 h. Water (900 mL) was added dropwise to the mixture and the excess of CBr_2_F_2_ was distilled in a dry ice trap. The residue was extracted with MTBE (*tert*-butyl-methyl ether) (7 × 100 mL), extract was washed with brine and dried with MgSO_4_. The solvent was distilled off at atmospheric pressure and the product was distilled in a vacuum. The yield was 29.4 g (37%); colorless liquid or solid; b.p. 80–81 °C (10 mbar); m.p. 14–17 °C. ^1^H-NMR (400 MHz, CDCl_3_) δ 8.27 (s, 1H, H_Py_), 8.35 (s, 1H, H_Py_). ^13^C-NMR (126 MHz, CDCl_3_) δ 111.5 (t, ^1^*J*_CF_ = 315.5 Hz, CBrF_2_), 135.2, 141.0, 145.9, 152.6. ^19^F-NMR (376.5 MHz, CDCl_3_) δ −18.4 (s, CBrF_2_). GC-MS, 70 eV, *m*/*z* (rel. int.): 258 (75) [M(^79^Br + ^35^Cl)]^+^, 260 (100) [M(^79^Br + ^37^Cl) or (^81^Br + ^35^Cl)]^+^, 262 (25) [M(^81^Br + ^37^Cl)]^+^. Anal. Calcd. for C_5_H_2_BrClF_2_N_2_O: C, 23.15; H, 0.78; N, 10.80; Found: C, 23.12; H, 0.68; N, 10.72.

### 3.3. 2-Chloro-5-trifluoromethoxypyrazine ***4***

Method A. The mixture of freshly sublimated SbF_3_ (103.7 g, 0.58 mol) and SbCl_5_ (17.4 g, 0.06 mol) was heated at 125–130 °C for 15 min and 2-chloro-5-(trichloromethoxy)pyrazine **2** (47.9 g, 0.19 mol) was added in portions at 100 °C to this mixture. The reaction mixture was stirred at 145–150 °C for 5 h and then 1 h at 155–160 °C. After cooling to r.t., the mixture was suspended in CH_2_Cl_2_ (700 mL), and solutions of K_2_CO_3_ (138 g, 1 mol) in 700 mL of water and KF (174 g, 3 mol) in 500 mL of water were carefully added to the mixture. The mixture was filtered through celite, the organic layer was separated, and the aqueous layer was extracted with CH_2_Cl_2_ (3 × 100 mL). The combined extracts were washed with water and dried with MgSO_4_. The solvent was distilled off at atmospheric pressure and the product was distilled in a vacuum.

Method B. Silver tetrafluoroborate (24.3 g, 0.125 mol) was added in portions to the solution of 2-(bromodifluoromethoxy)-5-chloropyrazine **3** (29.4 g, 0.11 mol) in CH_2_Cl_2_ (250 mL) at −78 °C. The mixture was warmed to r.t. and stirred for 24 h in darkness. The solution of sodium bicarbonate (11.4 g, 0.136 mol) in 150 mL of water was added to the reaction mixture and the mixture was filtered through celite. The organic layer was separated and the aqueous layer was extracted with CH_2_Cl_2_ (3 × 100 mL). The combined extracts were washed with water and dried with MgSO_4_. The solvent was distilled off at atmospheric pressure and the product was distilled in a vacuum.

The yield (Method A) was 26.0 g (68%); (Method B) 20.7 g (92%); colorless liquid; b.p. 80–81 °C (100 mbar). ^1^H-NMR (400 MHz, CDCl_3_) δ 8.23 (s, 1H, H_Py_), 8.29 (s, 1H, H_Py_). ^13^C-NMR (126 MHz, CDCl_3_) δ 119.5 (q, ^1^*J*_CF_ = 262.5 Hz, CF_3_), 134.6, 140.9, 145.8, 151.8. ^19^F-NMR (376.5 MHz, CDCl_3_) δ −57.6 (s, CF_3_). GC-MS, 70 eV, *m*/*z* (rel. int.): 198 (100) [M(^35^Cl)]^+^, 20 (30) [^37^Cl)]^+^. Anal. Calcd. for C_5_H_2_ClF_3_N_2_O: C, 30.25; H, 1.02; Cl, 17.86; N, 14.11; Found: C, 30.22; H, 0.98; Cl, 17.93; N, 14.09.

### 3.4. Reaction of 2-Chloro-5-trifluoromethoxypyrazine 4 with 3-Methylthiophenol and Alternative Route to 2-Chloro-5-fluoropyrazine ***5***

Method A. The mixture of 2-chloro-5-trifluoromethoxypyrazine **4** (1.0 g 5 mmol), 3-methylthiophenol (0.06 g, 0.5 mmol), and cesium carbonate (2.46 g, 7.5 mmol) in anhydrous DMF (5 mL) was stirred at r.t. under Argon for 24 h. The mixture was poured into water (20 mL) and extracted with ether (5 × 10 mL). The etheral solution was washed with 5% sodium bicarbonate solution and then with brine and dried with MgSO_4_. The solvent was distilled off at atmospheric pressure and the product was distilled in a vacuum.

Method B. The mixture of 2-chloro-5-trifluoromethoxypyrazine **4** (1.0 g 5 mmol), 3-methylthiophenol (0.62 g, 5 mmol), and cesium carbonate (2.46 g, 7.5 mmol) in anhydrous DMF (5 mL) was stirred at r.t. under Argon for 24 h. The mixture was poured into water (20 mL) and extracted with ether (5 × 10 mL). The etheral solution was washed with 5% sodium bicarbonate solution and then with brine and dried with MgSO_4_. The solvent was distilled off at atmospheric pressure and the volatile products were distilled into a liquid nitrogen trap in a high vacuum (0.2 mbar) at 35–40 °C (bath temperature). After redistillation at 100 mbar, 2-chloro-5-fluoropyrazine was obtained. The residue after the high vacuum distillation was separated by TLS chromatography using pentane/ethylacetate (5:1 by vol.) as an eluent, yielding a mixture of pirazines **6** and **7** (with ratio 1:2 by weight) and disubstituted pyrazine **8**.

Method C. The mixture of 2-chloro-5-trifluoromethoxypyrazine **4** (0.5 g 2.5 mmol), 3-methylthiophenol (0.93 g, 7.5 mmol), and cesium carbonate (2.46 g, 7.5 mmol) in anhydrous DMF (5 mL) was stirred at r.t. under Argon for 24 h. The mixture was poured into water (20 mL) and extracted with ether (5 × 10 mL). The etheral solution was washed with 5% sodium bicarbonate solution and then with brine and dried with MgSO_4_. The solvent was distilled off in a vacuum and the residue was purified by column chromatography using a mixture of hexane/ethylacetate (5:1 by vol.) as an eluent, yielding disubstituted pyrazine **8**.

An alternative route to 2-chloro-5-fluoropyrazine***5.*** To the solution of 2-amino-5-chloropyrazine (5 g, 38.6 mmol) in 50% aqueous HBF_4_ (20 mL), sodium nitrite (5.3 g, 77.2 mmol) was added in portions at 0–5 °C. The reaction mixture was gently heated to 18–20 °C and stirred for 4 h. The mixture was extracted with ether (5 × 20 mL), and the etheral solution was washed with brine and dried with MgSO_4_. The solvent was distilled off at atmospheric pressure and the product was distilled in a vacuum.

*2-Chloro-5-fluoropyrazine***5**. The yield was (Method A) 0.49 (74%), (method B) 0.12 g (18%), (alternative method) 4.77 g (93%); colorless liquid, b.p. 80–81 °C (100 mbar). ^1^H-NMR (500 MHz, CDCl_3_) δ 8.22 (s, 1H, H3′), 8.24 (d, 1H, *^3^J*_HF_ = 8.5 Hz, H6′). ^13^C-NMR (126 MHz, CDCl_3_) δ 132.4 (d, ^2^*J*_CF_ = 40.2 Hz, C6′), 141.2 (d, ^3^*J*_CF_ = 10.0 Hz, C3′), 145.3 (CCl), 159.3 (d, ^1^*J*_CF_ = 252.7 Hz, CF). ^19^F-NMR (376.5 MHz, CDCl_3_) δ −85 (s, CF). GC-MS, 70 eV, *m*/*z* (rel. int.): 132 (100) [M(^35^Cl)]^+^, 134 (32) [M(^37^Cl)]^+^. Anal. Calcd. for C_4_H_2_ClFN_2_: C, 36.25; H, 1.52; Cl, 26.75; N, 21.14; Found: C, 36.22; H, 1.53; Cl, 26.73; N, 21.09.

*The mixture of 2-chloro-5-(m-tolylthio)pyrazine***6***and 2-fluoro-5(m-tolylthio)pyrazine***7**. The yield of the mixture was 0.33 g: chloropyrazine 6–11%, fluoropyrazine 7–22% (the yield was determined by NMR and LC analyses of the mixture of pyrazines **6** and **7**). *Rf* = 0.8 pentane/ethylacetate (5:1). Chloropyrazine **6**: ^1^H-NMR (500 MHz, CDCl_3_) δ 2.36 (s, 3H, CH_3_), 7.25–7.27 (m, 1H, CH_Ar_), 7.34–7.37 (m, 1H, CH_Ar_), 7.40–7.42 (m, 2H, 2CH_Ar_), 7.96 (s, 1H, H6′_Py_), 8.37 (s, 1H, H6′_Py_); LC/MS (CI): *m*/*z* = 237 [M(^35^Cl) + H]^+^, 239 [M(^37^Cl) + H]^+^. Fluoropyrazine **7** ^1^H-NMR (500 MHz, CDCl_3_) δ 2.36 (s, 3H, CH_3_), 7.25–7.27 (m, 1H, CH_Ar_), 7.34–7.37 (m, 1H, CH_Ar_), 7.40–7.42 (m, 2H, 2CH_Ar_), 7.84 (s, 1H, H6′_Py_), 7.26 (d, 1H, *^3^J*_HF_ = 8.5 Hz, H3′_Py_). ^19^F-NMR (376.5 MHz, CDCl_3_) δ −87.9 (s, CF). LC/MS (CI): *m*/*z* = 221 [M + H]^+^.

*2,5-Bis(m-tolylthio)pyrazine***8**. Yield (method B) 0.4 g (25%), (method C) 0.75 g (93%); yellowish powder, m.p. 102–103 °C. *Rf* = 0.6 pentane/ethylacetate (5:1). ^1^H-NMR (400 MHz, CDCl_3_) δ 2.34 (s, 6H, 2CH_3_), 7.18–7.21 (m, 2H, 2CH_Ar_), 7.27 (t, 2H, ^3^*J*_HH_ = 7.6 Hz, 2*m-*CH_Ar_), 7.33–7.37 (m, 4H, 4CH_Ar_), 8.03 (s, 2H, CH_Py_). ^13^C-NMR (126 MHz, CDCl_3_) δ 21.3, 129.4, 129.6, 130.3, 131.7, 135.2, 139.7, 142.5, 153.6. LC/MS (CI): *m*/*z* = 325 [M + H]^+^. Calcd. for C_18_H_16_N_2_S_2_: C, 66.63; H, 4.97; N, 8.63; S, 19.76; Found: C, 66.68; H, 5.02; N, 8.59; S, 19.70.

### 3.5. tert-Butyl Methyl(5-(trifluoromethoxy)pyrazin-2-yl)carbamate ***9***

To the carefully degassed mixture of 2-chloro-5-trifluoromethoxypyrazine **4** (2 g, 10 mmol), *N*-Boc-methylamine (2 g, 15 mmol) and Cs_2_CO_3_ (6.5 g, 20 mmol) in anhydrous dioxane (30 mL) Pd_2_dba_3_ (0.09 g, 0.1 mmol) and bis[(2-diphenylphosphino)phenyl] ether (DPEphos) (0.05 g, 0.1 mmol) were added and the mixture was stirred at 100 °C for 12 h. After cooling to r.t., the mixture was diluted with water (20 mL), filtered through celite, and extracted with ethylacetate (5 × 10 mL). The organic extract was washed with brine, dried with MgSO_4_, and concentrated in a vacuum. After column chromatography with the mixture of hexane/ethylacetate (5:1 by vol.) as the eluent, pyrazine **9** was obtained. The yield was 2.4 g (82%); colorless powder, m.p. 78–79 °C. ^1^H-NMR (400 MHz, CDCl_3_) δ 1.54 (s, 9H, C(CH_3_)_3_), 3.39 (s, 3H, CH_3_), 8.18 (s, 1H, H_Py_), 8.82 (s, 1H, H_Py_). ^13^C-NMR (100 MHz, CDCl_3_) δ 28.2 (C(CH_3_)_3_), 33.8 (NCH_3_), 82.5 (C(CH_3_)_3_), 120.2 (q, ^1^*J*_CF_ = 262.5 Hz, CF_3_), 132.1, 137.6, 148.5, 150.1, 153.6. ^19^F-NMR (376.5 MHz, CDCl_3_) δ −57.5 (s, CF_3_). LC/MS (CI): *m*/*z* = 294 [M + H]^+^. Calcd. for C_11_H_14_F_3_N_3_O_3_: C, 45.05; H, 4.81; N, 14.33; Found: C, 45.08; H, 4.88; N, 14.29.

### 3.6. 5-(Trifluoromethoxy)pyrazine-2-carbonitrile ***10***

To the carefully degassed mixture of 2-chloro-5-trifluoromethoxypyrazine **4** (1.00 g, 5 mmol), Zn(CN)_2_ (0.39 g, 3.3 mmol) and Zn dust (0.6 g, 0.9 mmol) in anhydrous dimethylacetamide (10 mL) Pd_2_dba_3_ (0.14 g, 0.15 mmol) and *dppf* (0.17 g, 0.3 mmol) were added, and the mixture was stirred at 100 °C for 8 h. After cooling to r.t., the mixture was diluted with water (30 mL), filtered through celite, and extracted with ether (7 × 10 mL). The etheral solution was washed with brine (3 × 10 mL), dried with MgSO_4_, and the solvent was distilled off under atmospheric pressure. The residue was purified by TLC chromatography, yielding 0.34 g (36%) cyanopyrazine **10** as a colorless liquid with an intense almond scent, b.p. 95–96 °C (100 mbar). *Rf* = 0.8 pentane/ethylacetate (10:1). ^1^H-NMR (400 MHz, CDCl_3_) δ 8.51 (s, 1H, H_Py_), 8.62 (s, 1H, H_Py_). ^13^C-NMR (125.6 MHz, CDCl_3_) δ 114.5 (CN), 119.8 (q, *^1^J*_CF_ = 266.5 Hz, CF_3_), 127.5, 136.9, 145.7, 154.3. ^19^F-NMR (376.5 MHz, CDCl_3_) δ −57.5 (s, CF_3_). GC-MS, 70 eV, *m*/*z* (rel. int.): 189 (100) [M]^+^. Calcd. for C_6_H_2_F_3_N_3_O: C, 38.11; H, 1.07; N, 22.22; Found: C, 38.18; H, 1.16; N, 22.19.

### 3.7. Synthesis of Alkylpyrazines-Bearing Trifluoromethoxy Group by Kumada-Corriu Coupling

The solution of ethylmagnesium bromide (18 mL, 18 mmol, 1 mol/L solution in THF) or butylmagnesium chloride (9 mL, 18 mmol, 2 mol/L solution in THF) was added dropwise at 0 °C in an Argon atmosphere to the mixture of 2-chloro-5-trifluoromethoxypyrazine **4** (3.0 g, 15 mmol) and Fe(acac)_2_ (0.19 g, 0.75 mmol) in 30 mL of anhydrous THF. The mixture was warmed to r.t. and stirred for 3 h (in the case of ethylmagnesium bromide) or 4 h (in the case of butylmagnesium chloride). The reaction mixture was cooled to 0 °C and neutralized with 3% aqueous HCl. The product was extracted with ether (5 × 10 mL) and the etheral solution was washed with brine (5 × 10 mL) and dried with MgSO_4_. The solvent was distilled off at atmospheric pressure and the product was distilled in a vacuum.

*2-Ethyl-5-(trifluoromethoxy)pyrazine***11**. Yield 2.77 g (96%); colorless liquid with intense fruit scent, b.p. 83–85 °C (100 mbar). ^1^H-NMR (400 MHz, CDCl_3_) δ 1.31 (t, 3H, ^3^*J*_HH_ = 7.6 Hz, CH_3_), 2.84 (q, 2H, ^3^*J*_HH_ = 7.6 Hz, CH_2_) 8.13 (s, 1H, H_Py_), 8.35 (s, 1H, H_Py_). ^13^C-NMR (126 MHz, CDCl_3_) δ 13.3 (s, CH_3_), 22.8 (s, CH_2_), 120.1 (q, ^1^*J*_CF_ = 262.7 Hz, CF_3_), 135.0, 140.1, 151.8, 156.7. ^19^F-NMR (376.5 MHz, CDCl_3_) δ −57.3 (s, CF_3_). GC-MS, 70 eV, *m*/*z* (rel. int.): 192 (100) [M]^+^. Anal. Calcd. for C_7_H_7_F_3_N_2_O: C, 43.76; H, 3.67; N, 14.58; Found: C, 43.62; H, 3.53; N, 14.49.

*2-Butyl-5-(trifluoromethoxy)pyrazine***12**. 3.03 g (92%); colorless liquid with intense fruit scent, b.p. 83–85 °C (20 mbar). ^1^H-NMR (400 MHz, CDCl_3_) δ 0.92 (t, 3H, ^3^*J*_HH_ = 7.6 Hz, CH_3_), 1.37 (sext, 2H, ^3^*J*_HH_ = 7.6 Hz, CH_2_CH_3_), 1.67 (quint, 2H, ^3^*J*_HH_ = 7.6 Hz, CH_2_CH_2_CH_2_), 2.82 (t, 2H, ^3^*J*_HH_ = 7.6 Hz, CH_2_) 8.11 (s, 1H, H_Py_), 8.34 (s, 1H, H_Py_). ^13^C-NMR (126 MHz, CDCl_3_) δ 13.8 (s, CH_3_), 22.2 (s, CH_2_), 31.5 (s, CH_2_), 34.2 (s, CH_2_), 120.1 (q, ^1^*J_CF_* = 262.3 Hz, CF_3_), 135.0, 140.5, 151.8, 155.8. ^19^F-NMR (376.5 MHz, CDCl_3_) δ −57.3 (s, CF_3_). GC-MS, 70 eV, *m*/*z* (rel. int.): 220 (100) [M]+. Anal. Calcd. for C_9_H_11_F_3_N_2_O: C, 49.09; H, 5.04; N, 12.72; Found: C, 49.02; H, 5.03; N, 12.79.

### 3.8. 2-Phenyl-5-(trifluoromethoxy)pyrazine ***13***

Palladium acetate (34 mg, 0.15 mmol) and triphenylphosphine (158 mg, 0.6 mmol) were mixed together in carefully degassed dimethoxiethane (25 mL) and stirred for 15 min at r.t. under an Ar atmosphere. Chloropyrazine **4** (1.0 g, 5 mmol), phenylboronic acid (0.7 g, 6 mmol), and K_2_CO_3_ (1.4 g, 10 mmol) were added to the reaction mixture and the mixture was stirred at 75 °C for 20 h. After cooling to r.t., the mixture was diluted with water, filtered through celite, and extracted with *tret-*buthylmethyl ether (3 × 25 mL). The organic solution was washed with brine, dried with MgSO_4_, and evaporated in a vacuum. The residue was purified by column chromatography with hexane/ethylacetate (10:1 by vol.) as the eluent (*Rf* = 0.6). The yield was 0.95 g (80%); colorless powder, m.p. 80 °C. ^1^H-NMR (400 MHz, CDCl_3_) δ 7.48–7.55 (m, 3H, 3CH_Ph_), 7.92–8.00 (m, 2H, 2CH_Ph_), 8.50 (s, 1H, H_Py_), 8.70 (s, 1H, H_Py_). ^13^C-NMR (126 MHz, CDCl_3_) δ 120.2 (q, ^1^*J*_CF_ = 262.7 Hz, CF_3_), 126.8, 129.1, 130.0, 135.1, 135.2, 138.4, 150.9, 152.4. ^19^F-NMR (376.5 MHz, CDCl_3_) δ −57.2 (s, CF_3_). GC-MS, 70 eV, *m*/*z* (rel. int.): 240 (100) [M]^+^. Anal. Calcd. for C_11_H_7_F_3_N_2_O: C, 55.01; H, 2.94; N, 11.66; Found: C, 49.9; H, 3.00; N, 11.69.

### 3.9. Reaction of 2-Chloro-5-trifluoromethoxypyrazine ***4*** with Potassium Ethenyl Trifluoroborate

#### 3.9.1. 2-(Trifluoromethoxy)-5-vinylpyrazine **14**

To the carefully degassed mixture of 2-chloro-5-trifluoromethoxypyrazine **4** (2.0 g, 10 mmol), potassium ethenyl trifluoroborate (2.0 g, 15 mmol) and NEt_3_ (2 g, 20 mmol) in anhydrous *iso-*propanole (10 mL) PdCl_2_*dppf*·CH_2_Cl_2_ (0.4 g, 0.5 mmol) were added and the mixture was stirred for 20 h at 75 °C. After cooling to r.t., the mixture was diluted with water (20 mL), filtered through celite, and extracted with CH_2_Cl_2_ (5 × 10 mL). The organic extract was washed with 1% aqueous HCl, then with water, and dried with CaCl_2_. After the removal of the solvent at atmospheric pressure, the residue was distilled with deflegmator in a vacuum. The yield was 1.17 g (62%); colorless liquid with a pungent odor, b.p. 90–91 °C (100 mbar). ^1^H-NMR (500 MHz, CDCl_3_) δ 5.63 (d, 1H, ^3^*J*_HH_ = 11.0 Hz, =CH_2_), 6.33 (d, 1H, ^3^*J*_HH_ = 17.5 Hz, =CH_2_), 6.83 (dd, 1H, ^3^*J*_HH*-trans*_ = 17.5 Hz, ^3^*J*_HH*-cis*_ = 11.0 Hz, =CH_2_), 8.27 (s, 1H, H_Py_), 8.41 (s, 1H, H_Py_). ^13^C-NMR (100 MHz, CDCl_3_) δ 119.9 (q, ^1^*J*_CF_ = 262.7 Hz, CF_3_), 120.6, 131.7, 135.1, 139.1, 148.9, 152.1. ^19^F-NMR (376.5 MHz, CDCl_3_) δ −57.3 (s, CF_3_). GC-MS, 70 eV, *m*/*z* (rel. int.): 190 (100) [M]^+^. Anal. Calcd. for C_7_H_5_F_3_N_2_O: C, 44.22; H, 2.65; N, 14.73; Found: C, 44.29; H, 2.60; N, 14.69.

#### 3.9.2. 5-Divinylpyrazine **15**

The reaction was performed in the glass high pressure tube 50 mL vol. To the carefully degassed mixture of 2-chloro-5-trifluoromethoxypyrazine **4** (2.0 g, 10 mmol), potassium ethenyl trifluoroborate (5.4 g, 40 mmol) and NEt_3_ (4.1 g, 40 mmol) in anhydrous methanol (10 mL) PdCl_2_*dppf*·CH_2_Cl_2_ (0.8 g, 15 mmol) were added and the mixture was stirred for 20 h at 75 °C. After cooling to r.t., the mixture was diluted with water (20 mL), filtered through celite, and extracted with CH_2_Cl_2_ (5 × 10 mL). The organic extract was washed with 1% aqueous HCl, then with water, and dried with CaCl_2_. After the removal of the solvent at atmospheric pressure, the residue was purified by flash column chromatography with pentane/ethylacetate (5:1 by vol.) as the eluent (*Rf* = 0.8). The yield was 0.75 g (57%); colorless liquid, b.p. 45–47 °C (10 mbar). Compound **15** was partly polymerized under distillation and completely polymerized after 1-month storage in a refrigerator. ^1^H-NMR (500 MHz, CDCl_3_) δ 5.60 (d, 2H, ^3^*J*_HH_ = 11.0 Hz, 2 =CH_2_), 6.34 (d, 2H, ^3^*J*_HH_ = 17.5 Hz, =CH_2_), 6.83 (dd, 2H, ^3^*J*_HH*-trans*_ = 17.5 Hz, ^3^*J*_HH*-cis*_ =11.0 Hz, =CH_2_), 8.54 (s, 2H, H_Py_). ^13^C-NMR (100 MHz, CDCl_3_) δ 120.3, 133.3, 142.4, 149.5. GC-MS, 70 eV, *m*/*z* (rel. int.): 132 (100) [M]^+^. Anal. Calcd. for C_8_H_8_N_2_: C, 72.70; H, 6.10; N, 21.21; Found: C, 72.72; H, 6.10; N, 21.19.

### 3.10. 2-(Trifluoromethoxy)-5-((trimethylsilyl)ethynyl)pyrazine ***16***

To the carefully degassed mixture of 2-chloro-5-trifluoromethoxypyrazine **4** (3.0 g, 15 mmol), ethynyltrimethylsilane (4.4 g, 45 mmol) and NEt_3_ (9.2 g, 90 mmol) in anhydrous DMF (25 mL) PdCl_2_*dppf*·CH_2_Cl_2_ (0.62 g, 0.75 mmol) and CuI (0.14 g, 0.75 mmol) were added and the mixture was stirred for 20 h at r.t. The mixture was diluted with water (75 mL), filtered through celite, and extracted with MTBE (5 × 50 mL). The organic extract was washed with 1% aqueous HCl, then with brine, and dried with MgSO_4_. After the removal of the solvent, the residue was distilled in a vacuum. An effective deflegmator was necessary, due to difficulties in the separation of 1,4-bis(trimethylsilyl)buta-1,3-diyne that was formed as by-product. The yield was 3.0 g (77%); colorless oil, b.p. 118–120 °C (100 mbar). ^1^H-NMR (400 MHz, CDCl_3_) δ 0.26 (s, 9H, SiMe_3_), 8.35 (s, 2H, 2 H_Py_). ^13^C-NMR (100 MHz, CDCl_3_) δ-0.48 (s, SiMe_3_), 99.4 (s, C≡C), 100.2 (s, C≡C), 120.0 (q, ^1^*J*_CF_ = 263.6 Hz, CF_3_), 135.4 (s, C_Py_), 137.2 (s, C_Py_), 144.5 (s, C_Py_), 151.8 (s, C_Py_). ^19^F-NMR (376.5 MHz, CDCl_3_) δ −57.3 (s, CF_3_). GC-MS, 70 eV, *m*/*z* (rel. int.): 260 (100) [M]^+^. Anal. Calcd. for C_10_H_11_F_3_N_2_OSi: C, 46.14; H, 4.26; N, 10.76; Found: C, 46.19; H, 4.20; N, 10.69.

### 3.11. 2-Ethynyl-5-(trifluoromethoxy)pyrazine ***17***

The solution of 2-(trifluoromethoxy)-5-((trimethylsilyl)ethynyl)pyrazine **16** (2 g, 7.7 mmol) and 18-crown-6 (0.05 g, 0.2 mmol) in THF (20 mL) was added dropwise at 5–10 °C to the solution of K_2_CO_3_ (1.1 g, 7.7 mmol) in 3 mL of water. The mixture was stirred for 2 h at 10–15 °C, neutralized with 5% aqueous HCl, and extracted with MTBE (5 × 25 mL). The organic extract was washed with brine and dried with MgSO_4_. After the removal of the solvent at atmospheric pressure, the residue was distilled in a vacuum. The yield was 1.2 g (85%); colorless oil or solid with a grass scent, b.p. 76–77 °C (10 mbar), m.p. 45–46 °C. ^1^H-NMR (400 MHz, CDCl_3_) δ 3.33 (s, 1H, ≡CH), 8.39 (s, 2H, 2 H_Py_). ^13^C-NMR (100 MHz, CDCl_3_) δ 78.8 (s, ‒C≡), 81.4 (s, ≡CH), 119.9 (q, ^1^*J*_CF_ = 263.6 Hz, CF_3_), 135.6 (s, C_Py_), 136.4 (s, C_Py_), 144.8 (s, C_Py_), 152.2 (s, C_Py_). ^19^F-NMR (376.5 MHz, CDCl_3_) δ −57.3 (s, CF_3_). GC-MS, 70 eV, *m*/*z* (rel. int.): 188 (100) [M]^+^. Anal. Calcd. for C_7_H_3_F_3_N_2_O: C, 44.70; H, 1.61; N, 14.89; Found: C, 44.79; H, 1.60; N, 14.88.

### 3.12. 2-Ethynyl-5-methoxypyrazine ***18***

Method A. The solution of 2-(trifluoromethoxy)-5-((trimethylsilyl)ethynyl)pyrazine **16** (1 g, 3.8 mmol) in anhydrous methanol (3 mL) was added to 5 mL of the methanolic solution of KOH (0.43 g, 7.7 mmol) at 0 °C. The mixture was stirred for 2 h at 15 °C and the solvent was removed in a vacuum (without heating). The residue was suspended in ether and neutralized with 3% aqueous HCl. The organic solution was separated and the aqueous layer was extracted with ether (3 × 10 mL). The combined organic solutions were washed with brine and dried with MgSO_4_. After the removal of the solvent at atmospheric pressure, the residue was distilled in a vacuum.

Method B. The solution of 2-Ethynyl-5-(trifluoromethoxy)pyrazine **17** (1 g, 5.3 mmol) in anhydrous methanol (3 mL) was added to 5 mL of the methanolic solution of KOH (0.43 g, 7.7 mmol) at −10 °C. The mixture was stirred for 2 h at 15 °C and the solvent was removed in a vacuum (without heating). The residue was suspended in ether and neutralized with 3% aqueous HCl. The organic solution was separated and the aqueous layer was extracted with ether (3 × 10 mL). The combined organic solutions were washed with brine and dried with MgSO_4_. After the removal of the solvent at atmospheric pressure, the residue was distilled in a vacuum.

The yield (Method A) was 0.34 g (65%), (Method B) 0.63 g (88%); colorless solid, m.p. 40–41 °C, b.p. 88–90 °C (10 mbar); ^1^H-NMR (400 MHz, CDCl_3_) δ 3.22 (s, 1H, ≡CH), 3.98 (s, 3H, CH_3_), 8.18 (s, 2H, 2 H_Py_), 8.25 (s, 2H, 2 H_Py_). ^13^C-NMR (100 MHz, CDCl_3_) δ 53.9 (s, CH_3_), 78.9 (s, ≡CH), 80.0 (s, ‒C≡), 130.4 (s, C_Py_), 135.6 (s, C_Py_), 144.4 (s, C_Py_), 159.4 (s, C_Py_). GC-MS, 70 eV, *m*/*z* (rel. int.): 134 (100) [M]^+^. Anal. Calcd. for C_7_H_6_N_2_O: C, 62.68; H, 4.51; N, 20.88; Found: C, 62.62; H, 4.44; N, 20.80.

### 3.13. 1-(5-(Trifluoromethoxy)pyrazin-2-yl)ethan-1-one ***19***

Mercury oxide (0.38 g, 1.8 mmol) was added to the mixture of trifluoroacetic acid (60 mL, 800 mmol) and water (6 mL, 332 mmol) and stirred until dissolving (apr 10 min). The mixture was cooled to 10 °C and 2-ethynyl-5-(trifluoromethoxy)pyrazine **17** (1.0 g, 5.3 mmol) was added. The mixture was stirred at 40 °C for 1 h and trifluoroacetic acid was removed in a vacuum (300 mbar). Water (20 mL) was added to the residue and the mixture was neutralized with NaHCO_3_ to pH 6. The product was extracted with CHCl_3_ (3 × 10 mL) and the extract was washed with water and dried with MgSO_4_. After the removal of the solvent at atmospheric pressure, the residue was distilled in a vacuum. The yield was 0.92 g (84%); colorless solid, m.p. 44–45 °C, b.p. 94–95 °C (10 mbar); ^1^H-NMR (400 MHz, CDCl_3_) δ 2.69 (s, 3H, CH_3_), 8.42 (s, 2H, 2 H_Py_), 8.92 (s, 2H, 2 H_Py_). ^13^C-NMR (100 MHz, CDCl_3_) δ 25.9 (s, CH_3_), 119.9 (q, ^1^*J*_CF_ = 264.6 Hz, CF_3_), 134.1 (s, C_Py_), 141.3 (s, C_Py_), 145.6 (s, C_Py_), 155.1 (s, C_Py_), 197.5 (s, CO). ^19^F-NMR (376.5 MHz, CDCl_3_) δ −57.3 (s, CF_3_). GC-MS, 70 eV, *m*/*z* (rel. int.): 206 (100) [M]^+^. Anal. Calcd. for C_7_H_5_F_3_N_2_O: C, 40.79; H, 2.45; N, 13.59; Found: C, 40.70; H, 2.44; N, 13.60.

## 4. Conclusions

It was shown that 2-chloro-5-trifluoromethoxypyrazine is suitable for further transformations via chlorine atom substitution in Buchwald-Hartwig amination, Kumada-Corriu, and Suzuki and Sonogashira coupling reactions. At the same time, the trifluoromethoxy group attached to the electron-deficient pyrazine ring was easily destructed and displaced under the action of *O-* or *S-* nucleophiles. This property must be taken into account, especially in the processes of bioactive molecules modeling. On the other hand, this fact opens up the possibility to explore trifluoromethoxy moiety as a leaving group for nucleophilic substitution.

Efficient and scalable methods for 2-chloro-5-trifluoromethoxypyrazine synthesis, developed in this work, and its synthetic profile allow us to consider this molecule as perspective for wide synthetic utility.

## Supplementary Materials

^1^H-, ^13^C-, and ^19^F-NMR spectra of products associated with this article are available online.
