# Supplementary material for: Trifluoromethoxypyrazines: Preparation and Properties
Source: Molecules. 2020 May 9;25(9):2226. doi: 10.3390/molecules25092226 (PMC7248996; doi:10.3390/molecules25092226)
Supplement: Supplementary file 1 [file molecules-25-02226-s001.pdf]

Supplementary Materials

Trifluoromethoxypyrazines: Preparation and Properties

Taras M. Sokolenko [1], Yurii L. Yagupolskii [1,*]

[1] Institute of Organic Chemistry, NAS of Ukraine, Murmans'ka St. 5, Kyiv 02660, Ukraine
* Correspondence: Yagupolskii@ioch.kiev.ua; Tel.: +380-44-559-03-49

Received: date; Accepted: date; Published: date

1.1. 2-Chloro-5-(trichloromethoxy)pyrazine ***2*** *(^{1}H NMR).*

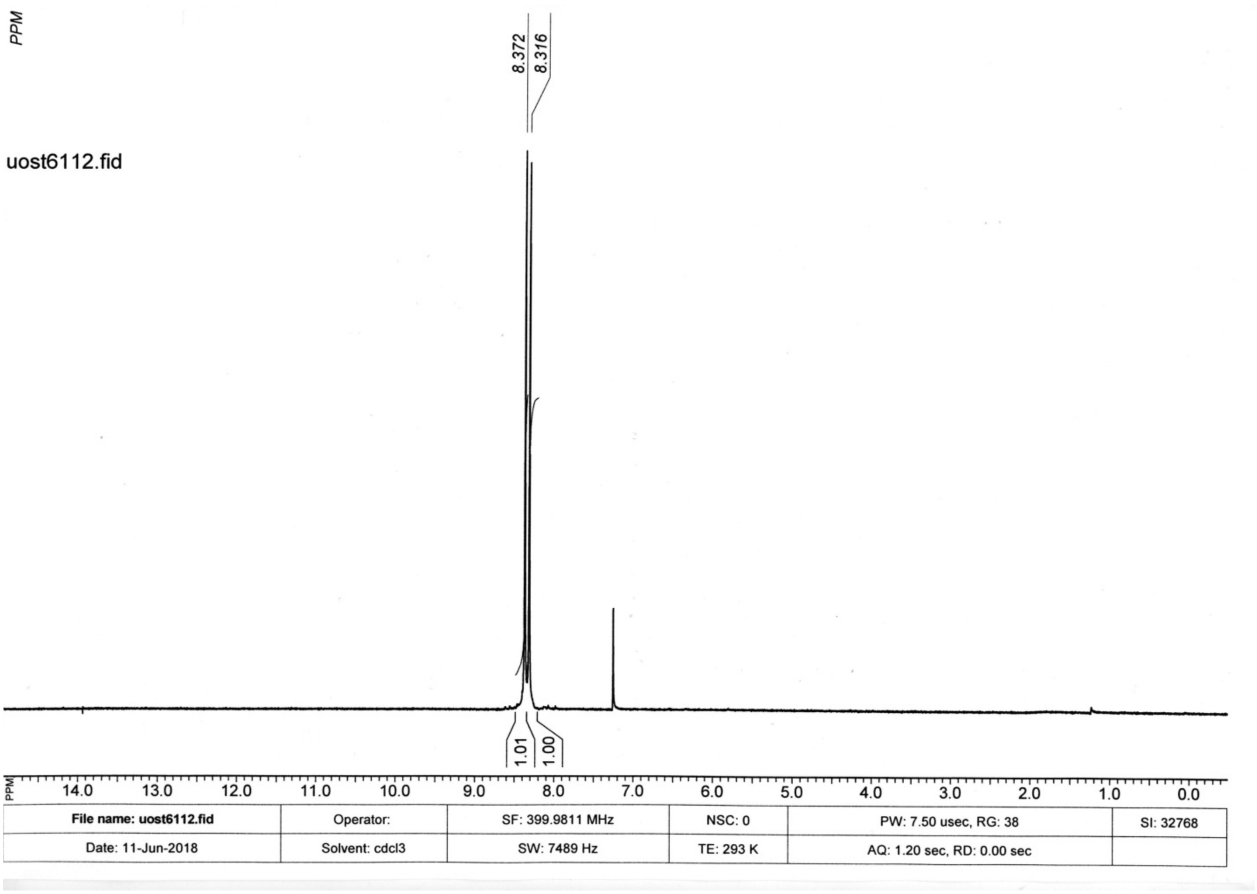

1.2. 2-Chloro-5-(trichloromethoxy)pyrazine ***2*** *(^{13}C NMR).*

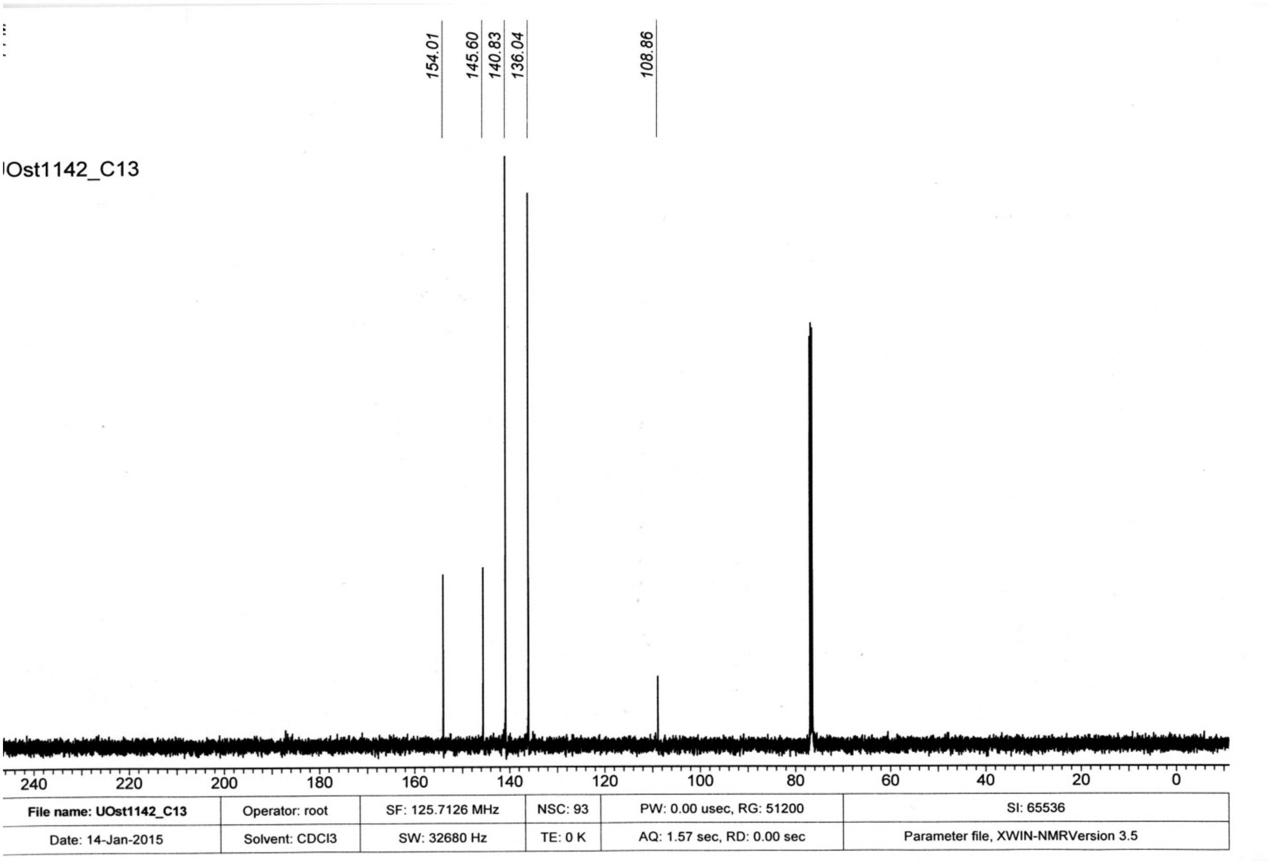

2.1. 2-(Bromodifluoromethoxy)-5-chloropyrazine **3** (^{1}H NMR).

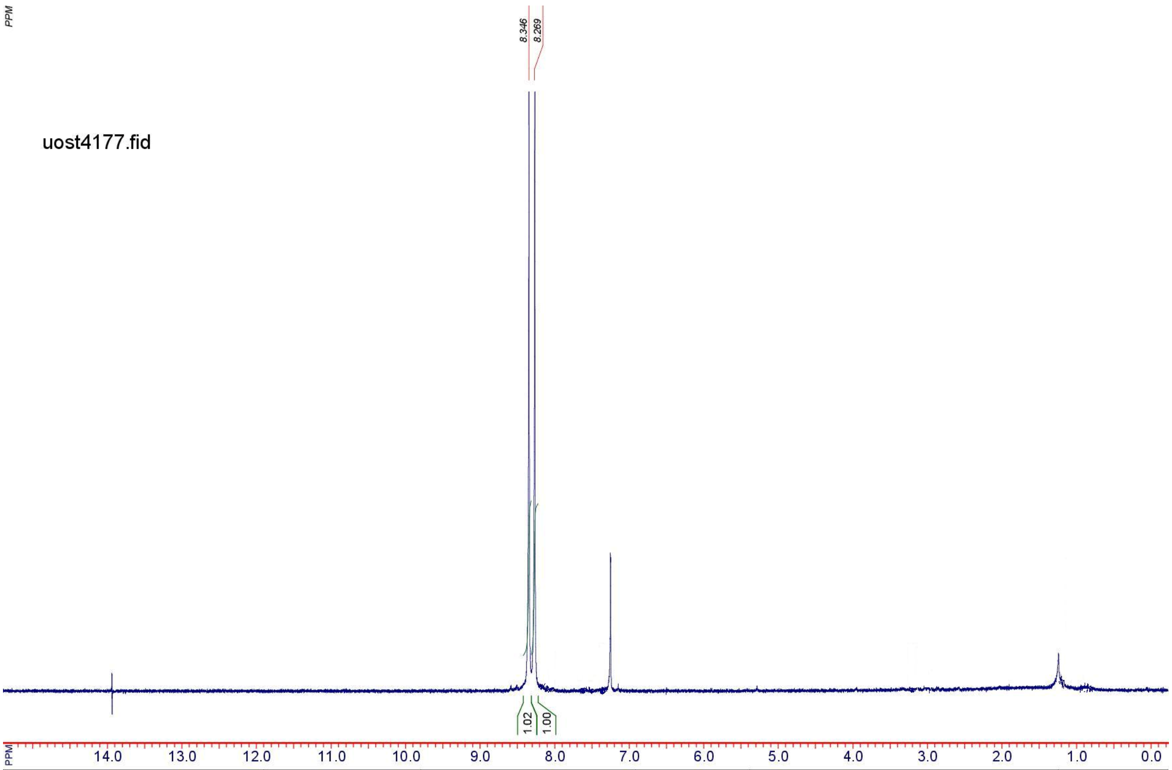

File name: uost4177.fid	Operator:	SF: 399.9811 MHz	NSC: 0	PW: 7.50 usec, RG: 30	SI: 32768
Date: 18-Apr-2018	Solvent: cdcl3	SW: 7489 Hz	TE: 293 K	AQ: 1.20 sec, RD: 0.00 sec	

2.2. 2-(Bromodifluoromethoxy)-5-chloropyrazine **3** (^{13}C NMR)

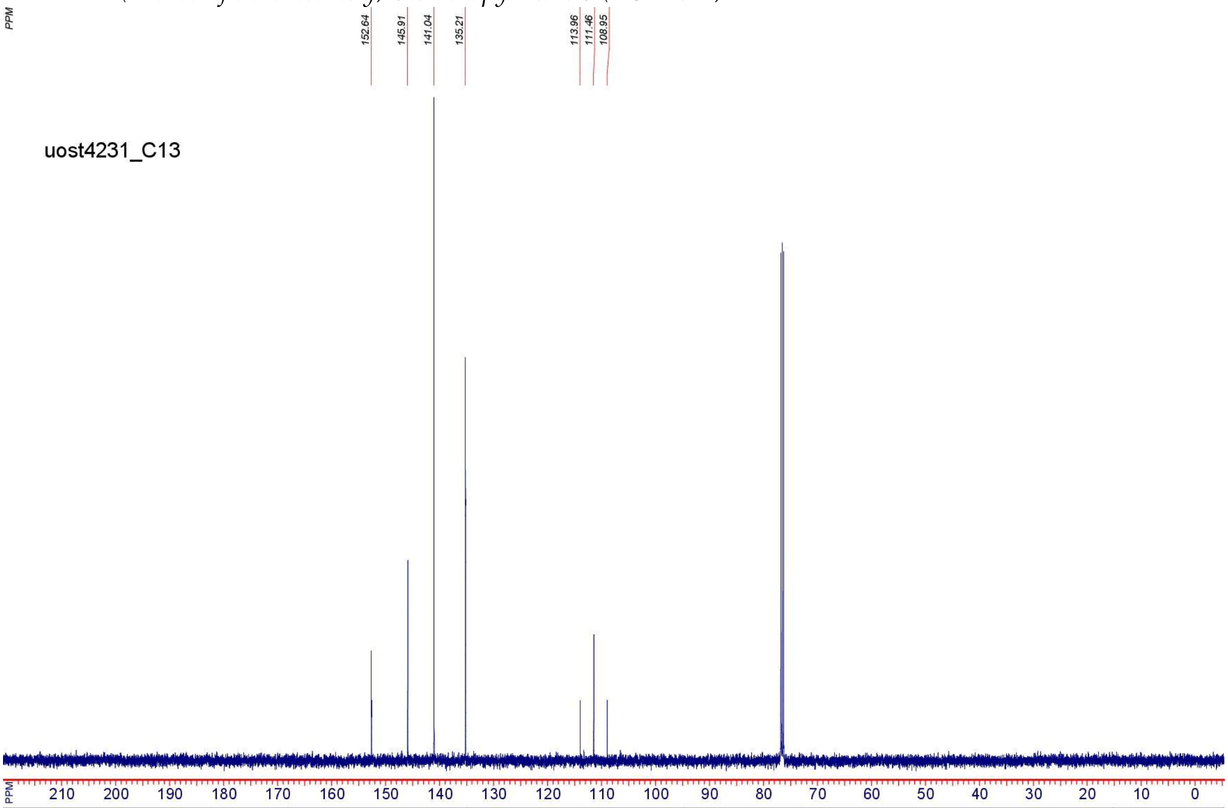

File name: uost4231_C13	Operator: root	SF: 125.6935 MHz	NSC: 115	PW: 0.00 usec, RG: 51200	SI: 65536
Date: 27-Apr-2018	Solvent: CDCl3	SW: 32680 Hz	TE: 683 K	AQ: 1.57 sec, RD: 0.00 sec	

2.3. 2-(Bromodifluoromethoxy)-5-chloropyrazine **3** (^{19}F NMR).

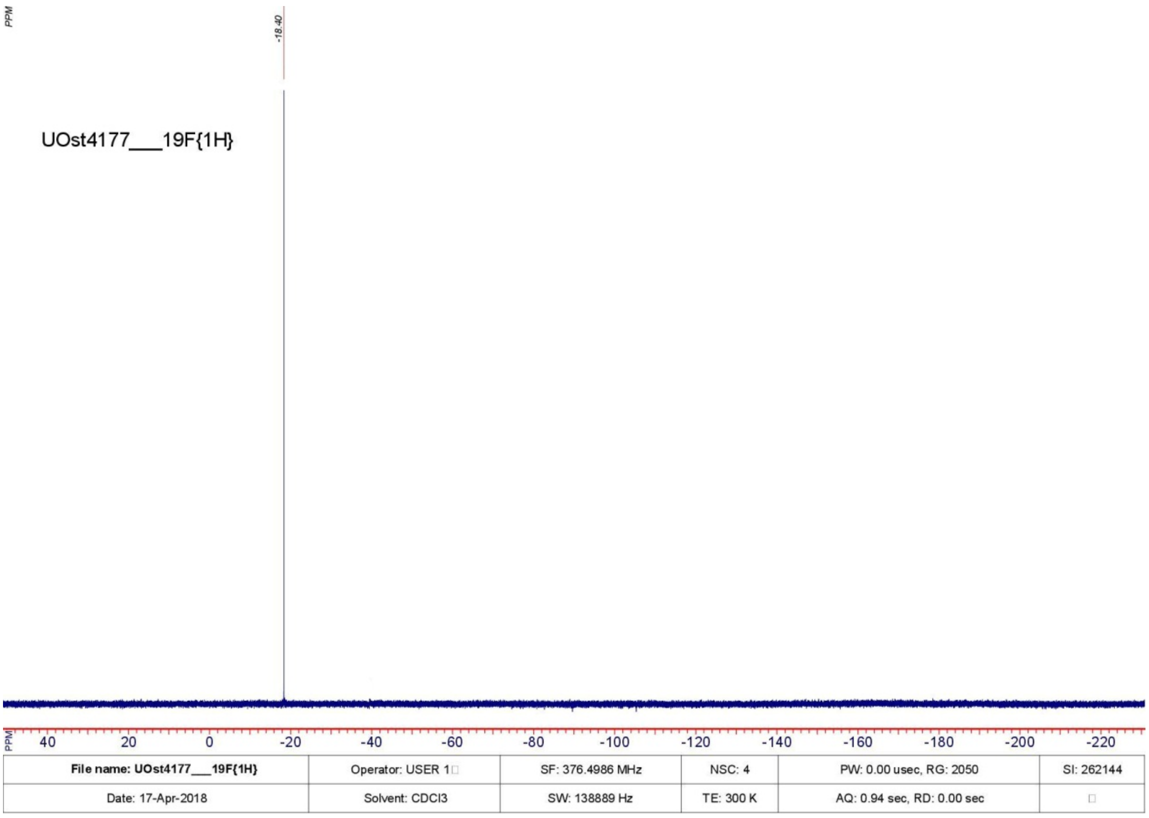

*3.1. 2-Chloro-5-trifluoromethoxypyrazine **4** (^{1}H NMR).*

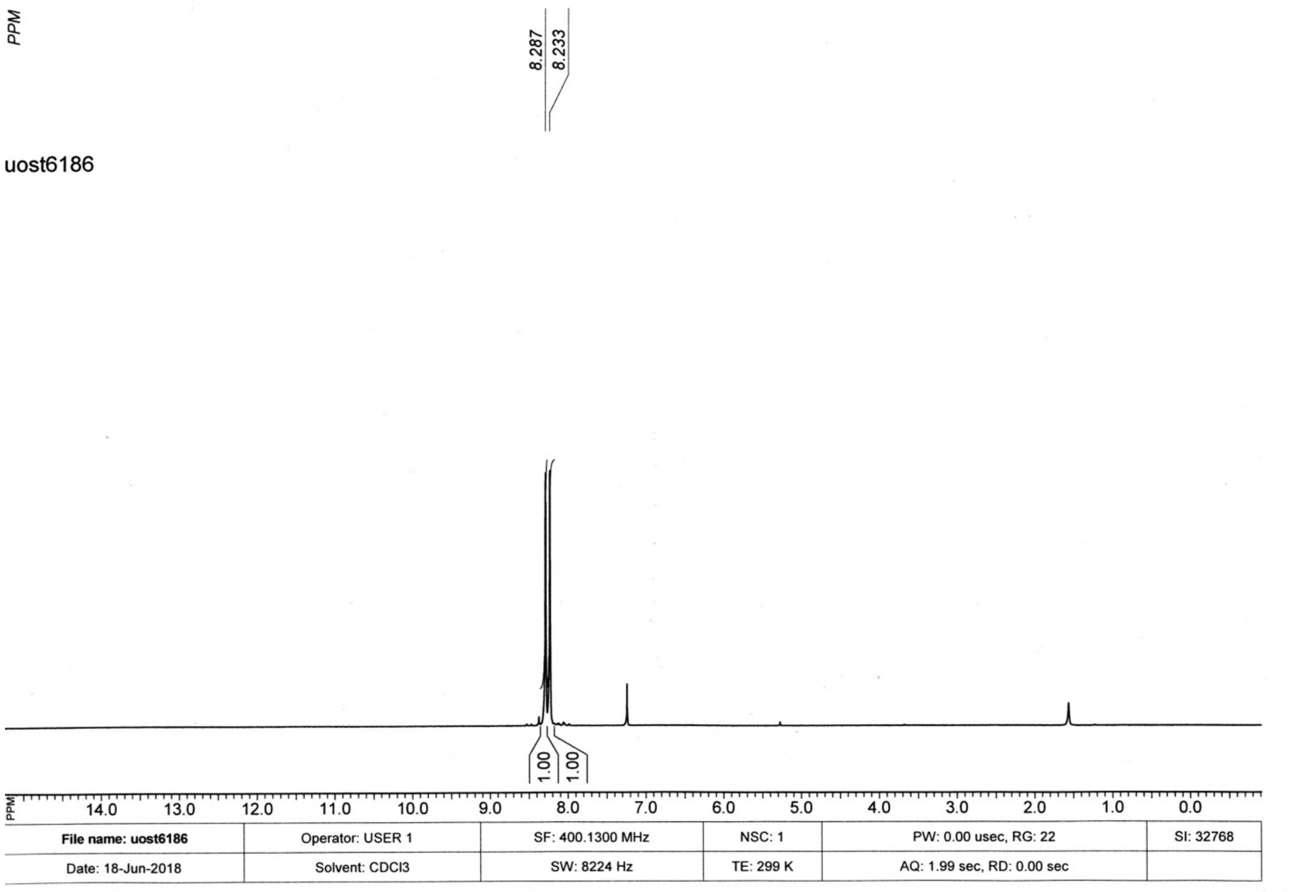

3.2. *2-Chloro-5-trifluoromethoxypyrazine **4** (^{13}C NMR).*

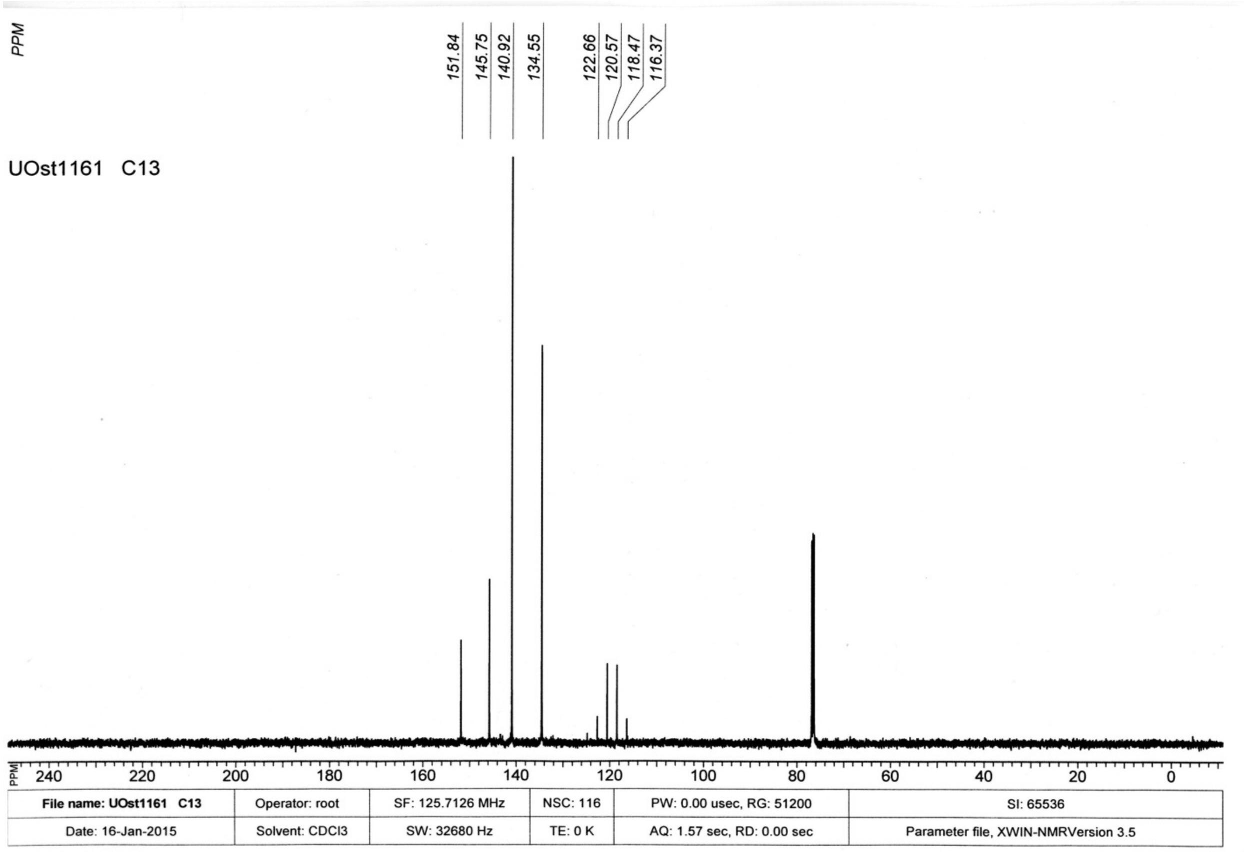

3.3. *2-Chloro-5-trifluoromethoxypyrazine **4** (^{19}F NMR).*

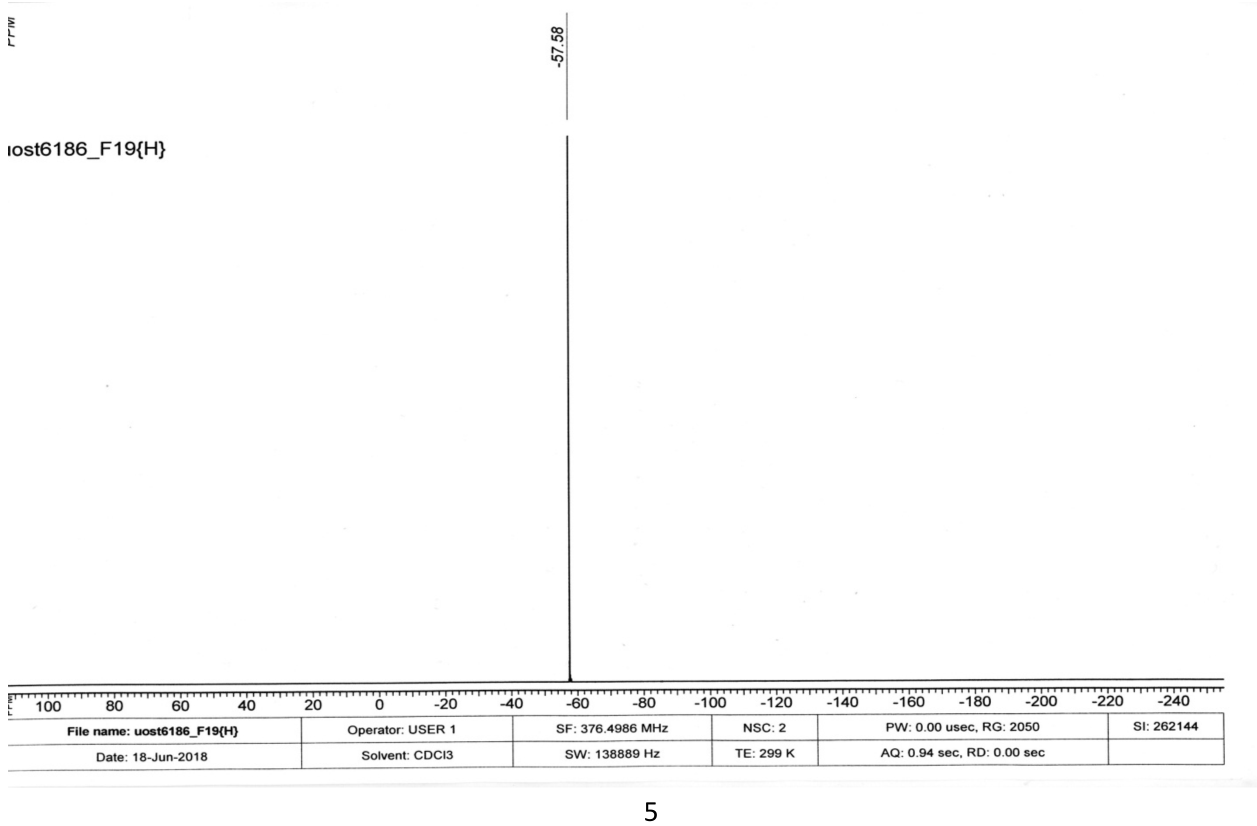

4.1. 2-Chloro-5-fluoropyrazine **5** (^{1}H NMR).

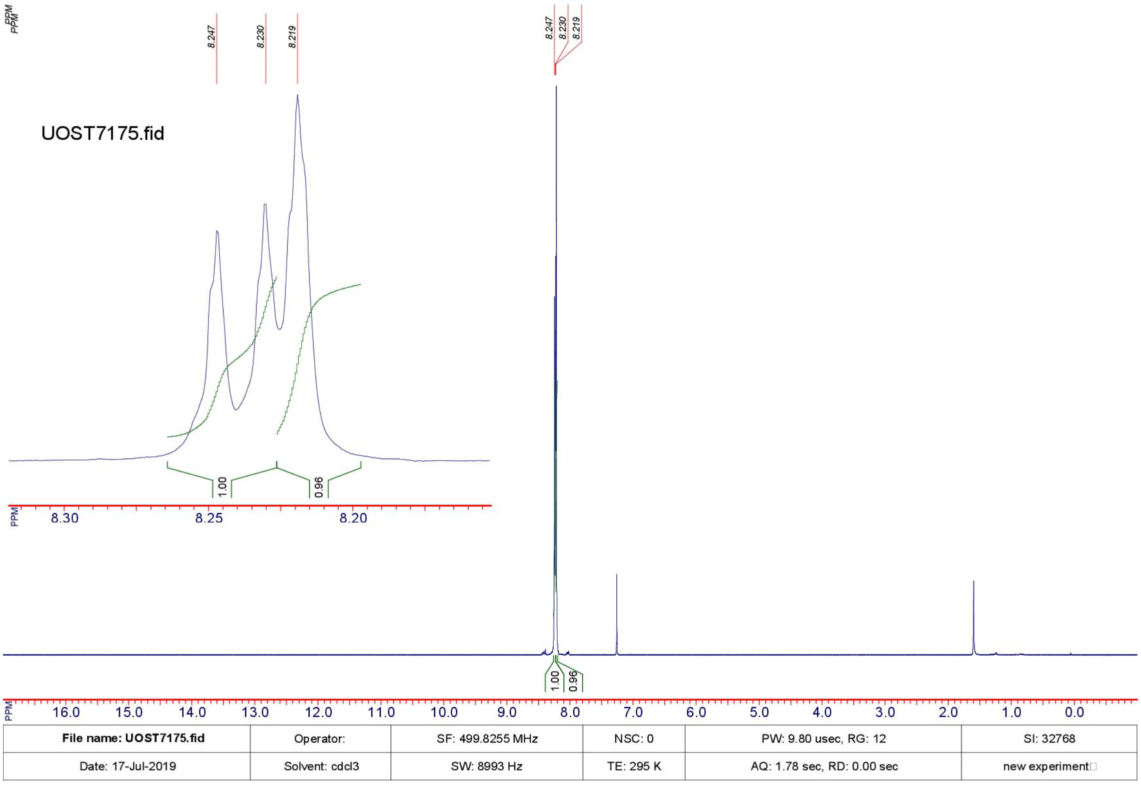

4.2. 2-Chloro-5-fluoropyrazine **5** (^{13}C NMR).

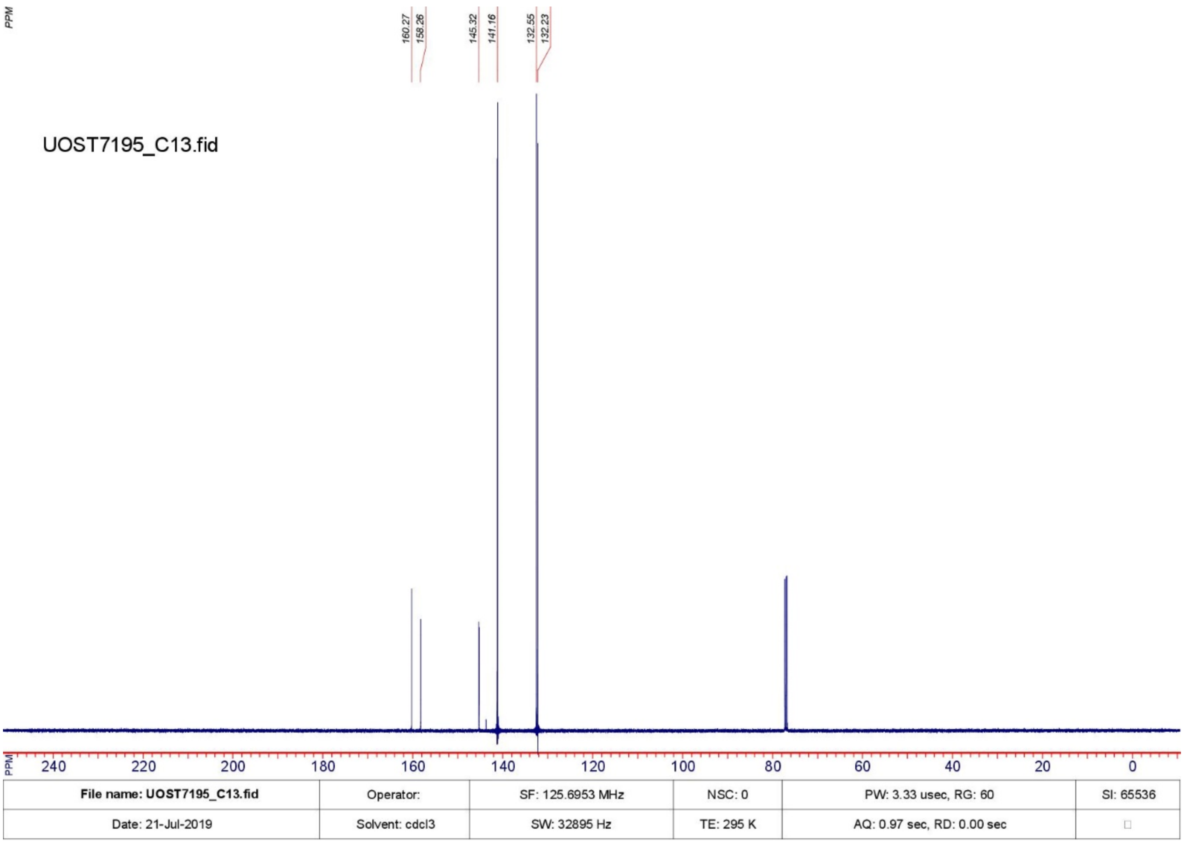

4.3. *2-Chloro-5-fluoropyrazine **5** (^{19}F NMR).*

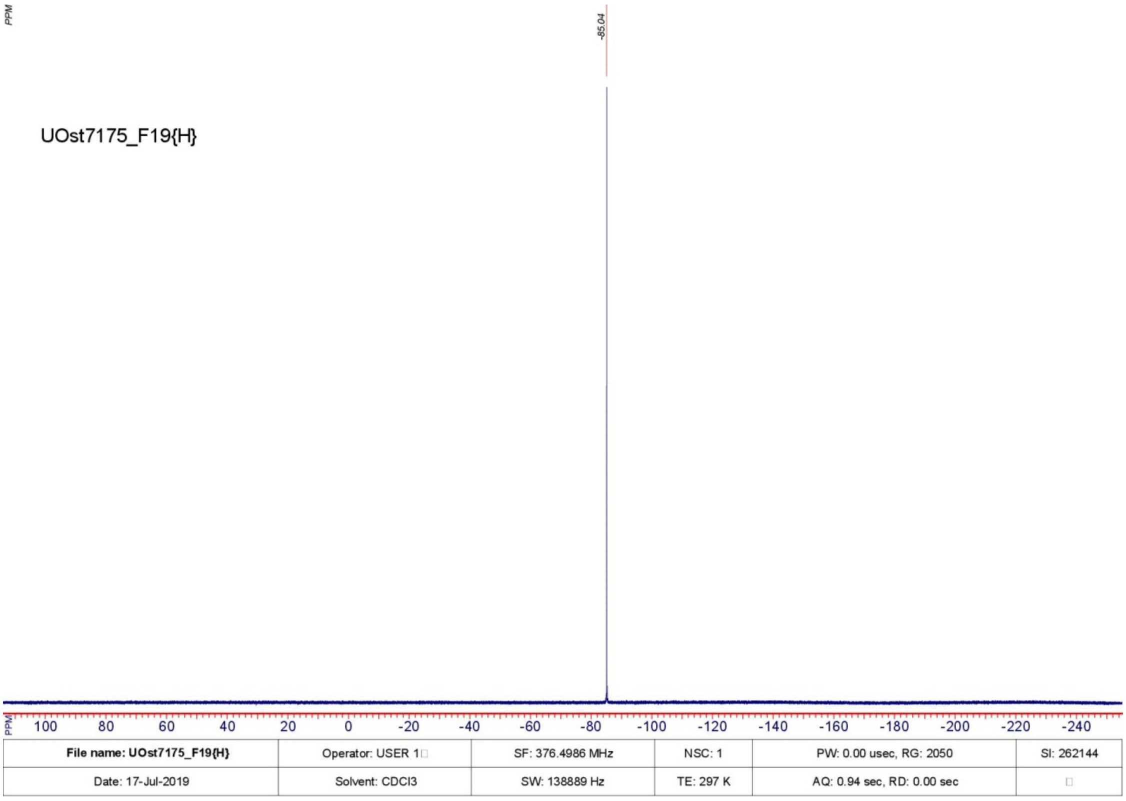

5.1. *The mixture of 2-chloro-5-(m-tolylthio)pyrazine **6** and 2-fluoro-5(m-tolylthio)pyrazine **7** (^{1}H NMR).*

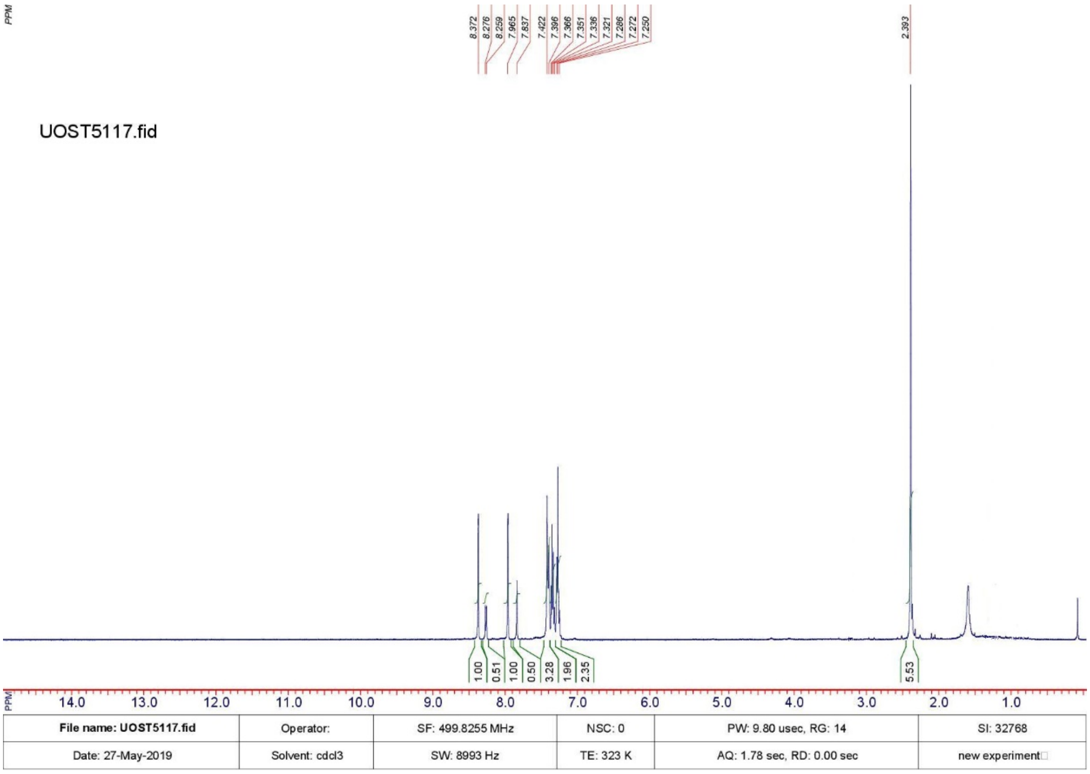

5.2. *The mixture of 2-chloro-5-(m-tolylthio)pyrazine **6** and 2-fluoro-5(m-tolylthio)pyrazine **7** (^{19}F NMR).*

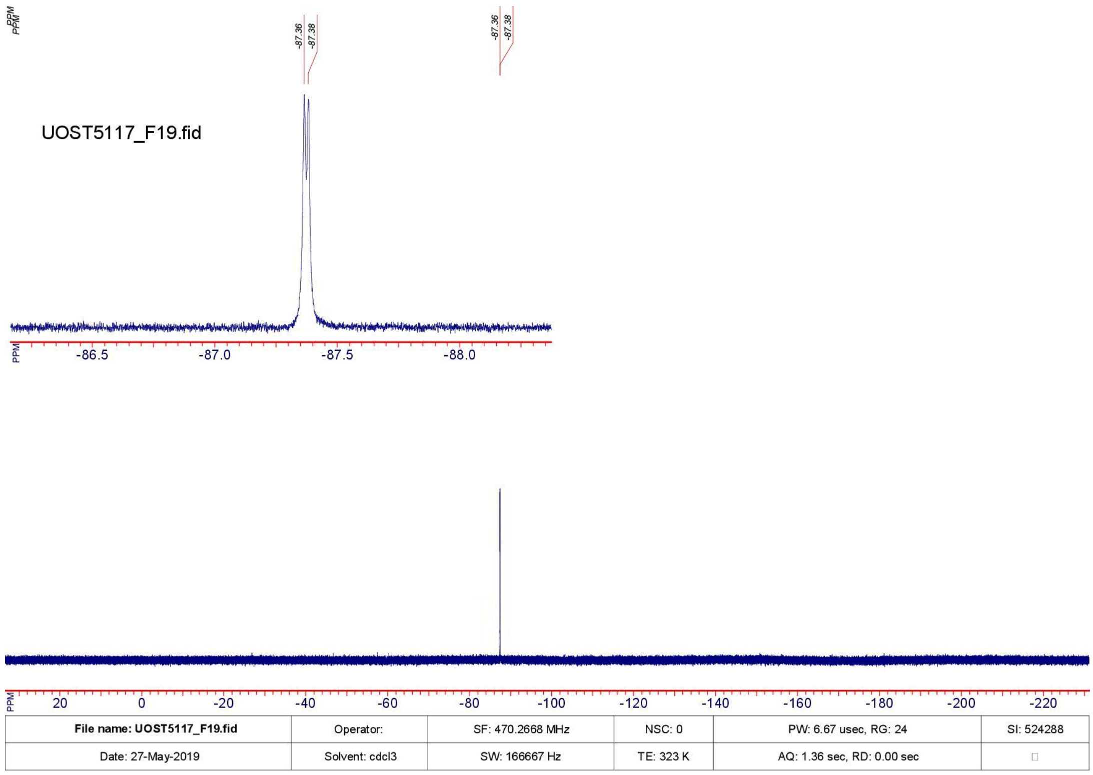

6.1. *2,5-Bis(m-tolylthio)pyrazine **8** (^{1}H NMR).*

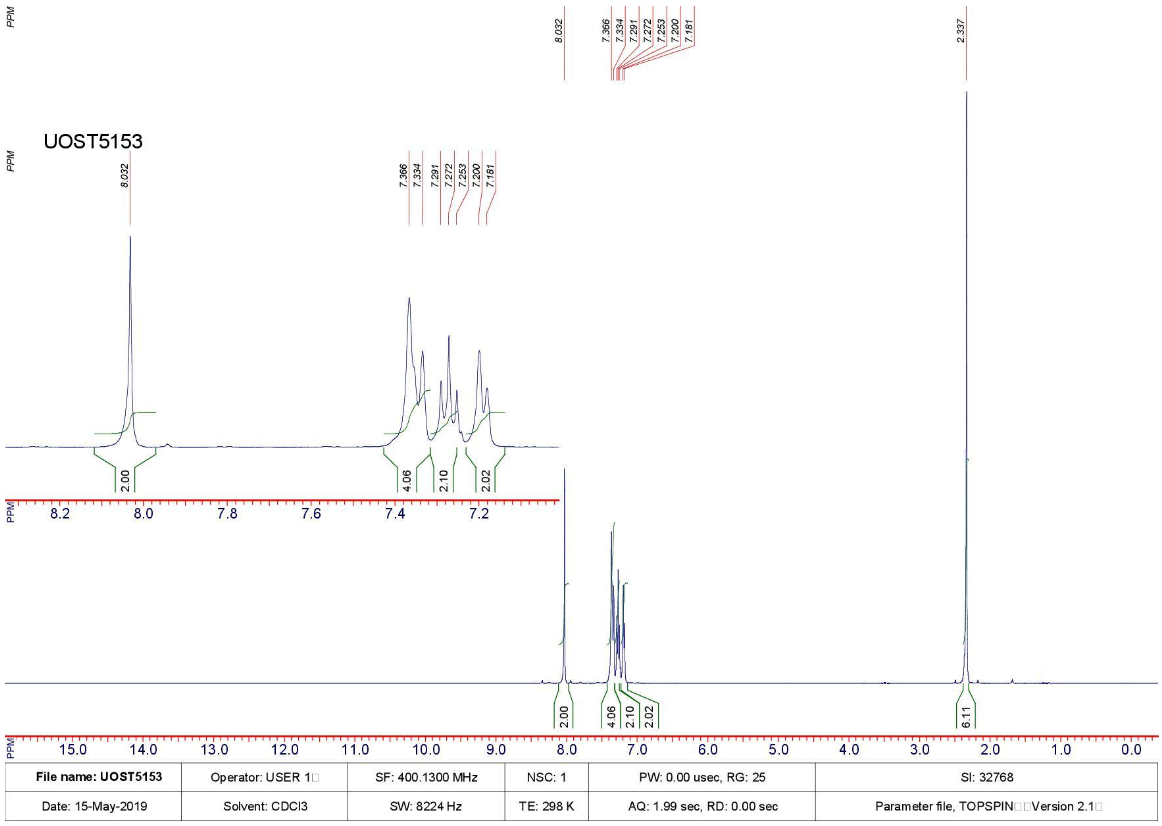

*6.2.   2,5-Bis(m-tolylthio)pyrazine **8** (^{13}C NMR).*

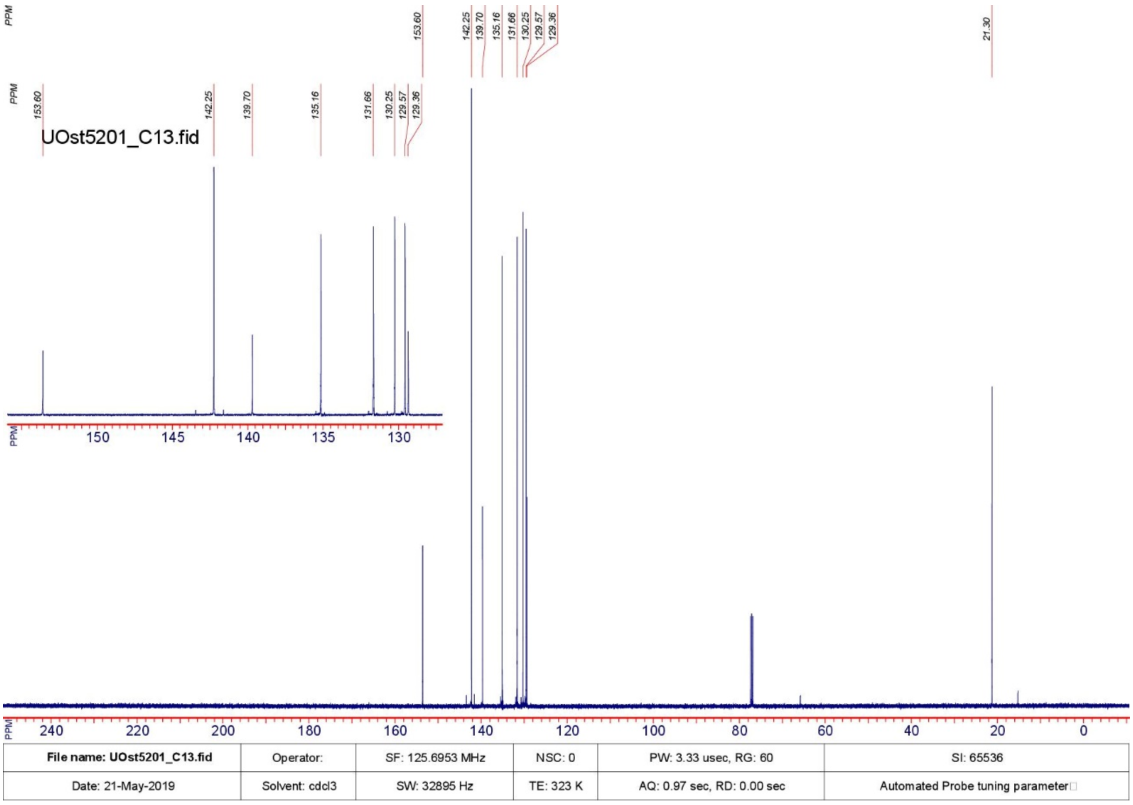

*7.1.   tert-Butyl methyl(5-(trifluoromethoxy)pyrazin-2-yl)carbamate **9** (^{1}H NMR).*

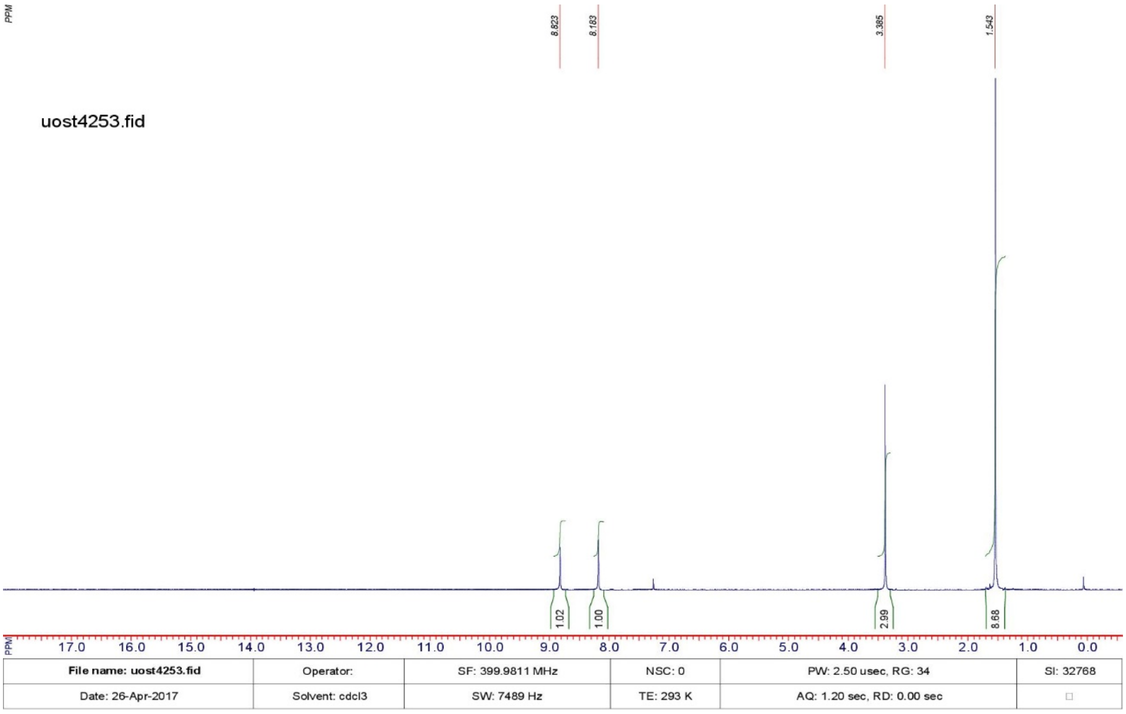

7.2. *tert-Butyl methyl(5-(trifluoromethoxy)pyrazin-2-yl)carbamate* ***9*** *(^{13}C NMR).*

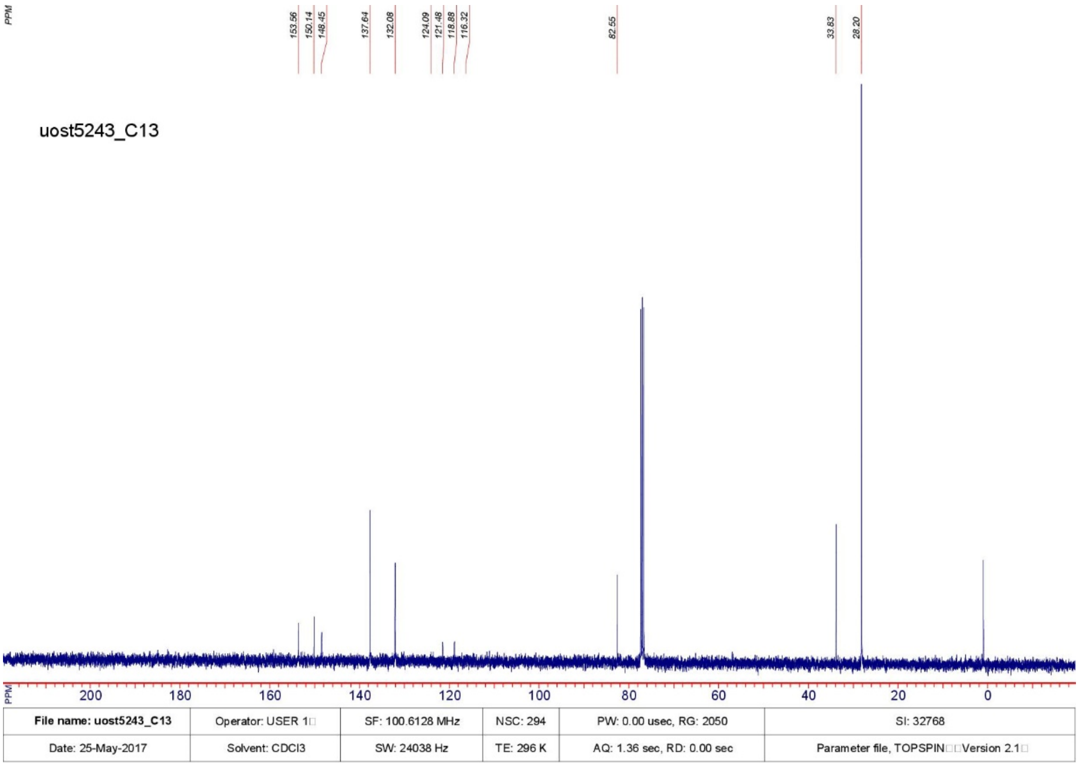

7.3. *tert-Butyl methyl(5-(trifluoromethoxy)pyrazin-2-yl)carbamate* ***9*** *(^{19}F NMR).*

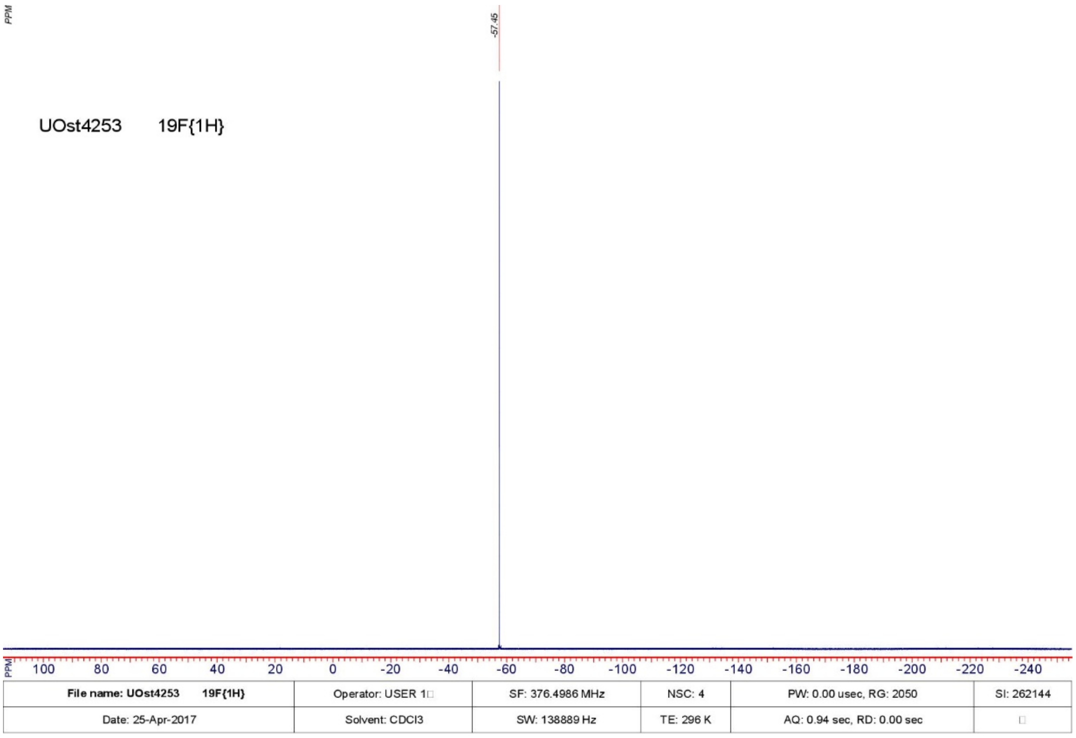

*8.1. 5-(Trifluoromethoxy)pyrazine-2-carbonitrile **10** (^{1}H NMR).*

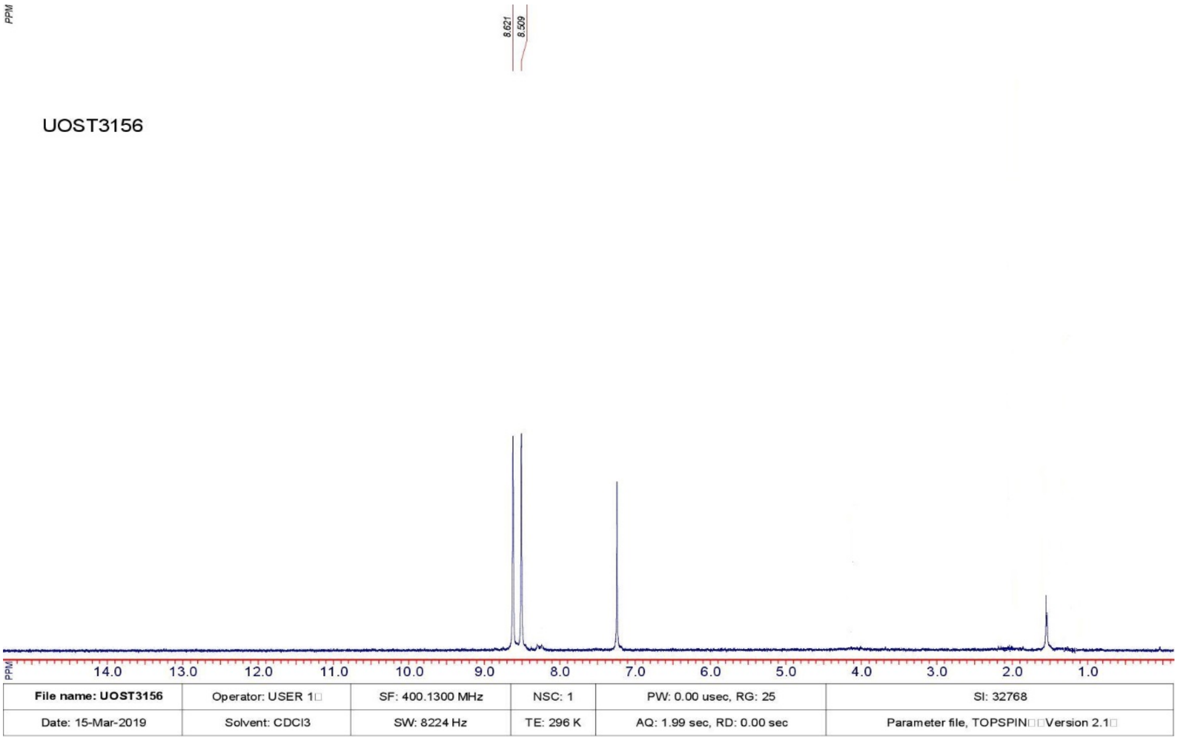

*8.2. 5-(Trifluoromethoxy)pyrazine-2-carbonitrile **10** (^{13}C NMR).*

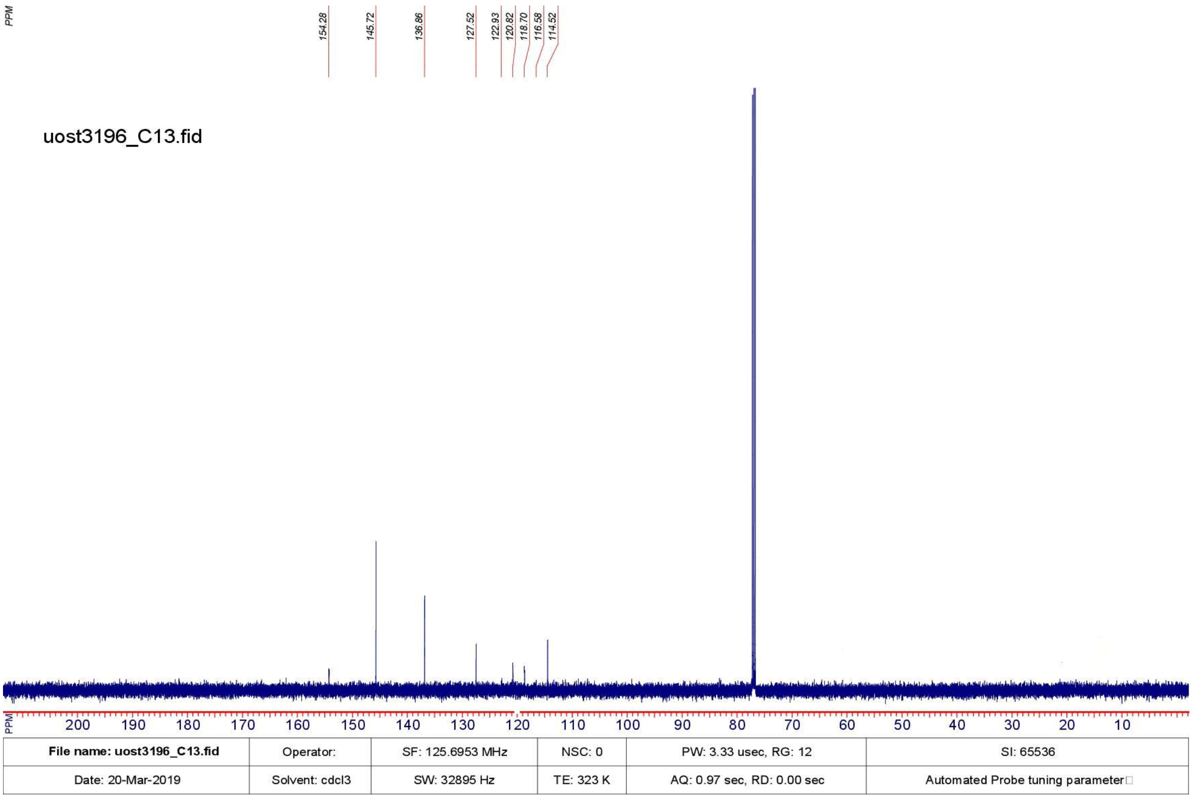

8.3. 5-(Trifluoromethoxy)pyrazine-2-carbonitrile ***10*** *(^{19}F NMR).*

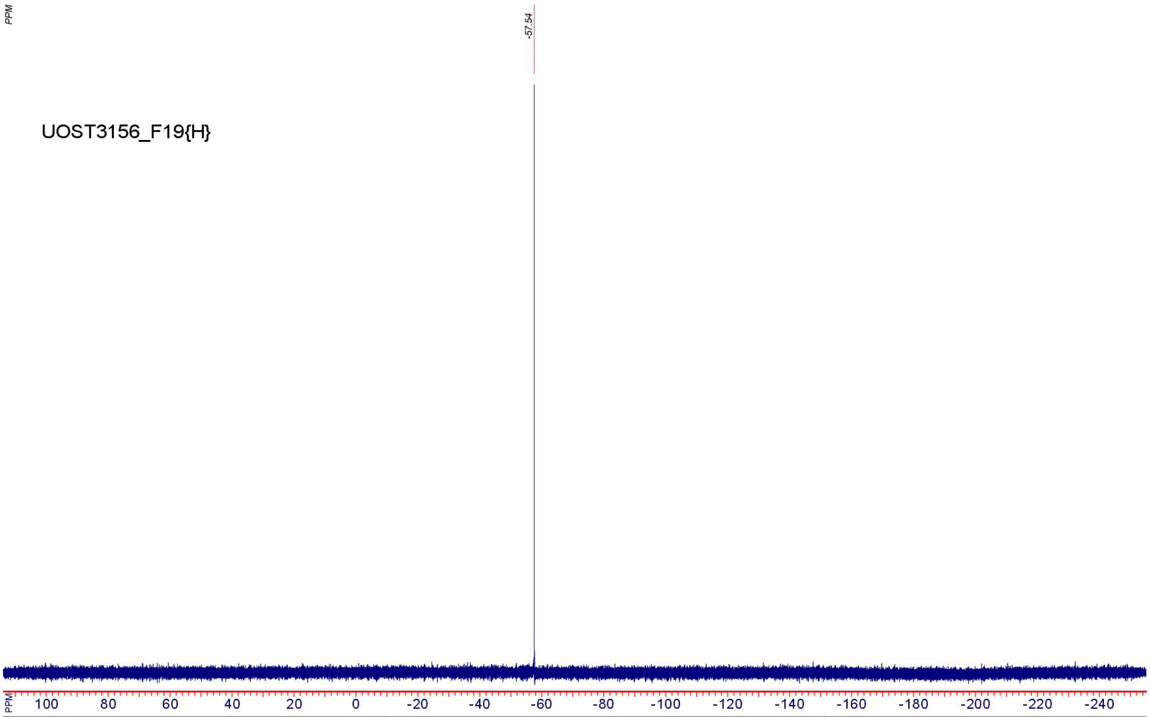

**File name: UOST3156_F19{H}**	Operator: USER 1	SF: 376.4986 MHz	NSC: 2	PW: 0.00 usec, RG: 724	SI: 262144
Date: 15-Mar-2019	Solvent: CDCl3	SW: 138889 Hz	TE: 297 K	AQ: 0.94 sec, RD: 0.00 sec	Parameter file, TOPSPIN Version 2.1

9.1. 2-Ethyl-5-(trifluoromethoxy)pyrazine ***11*** *(^{1}H NMR).*

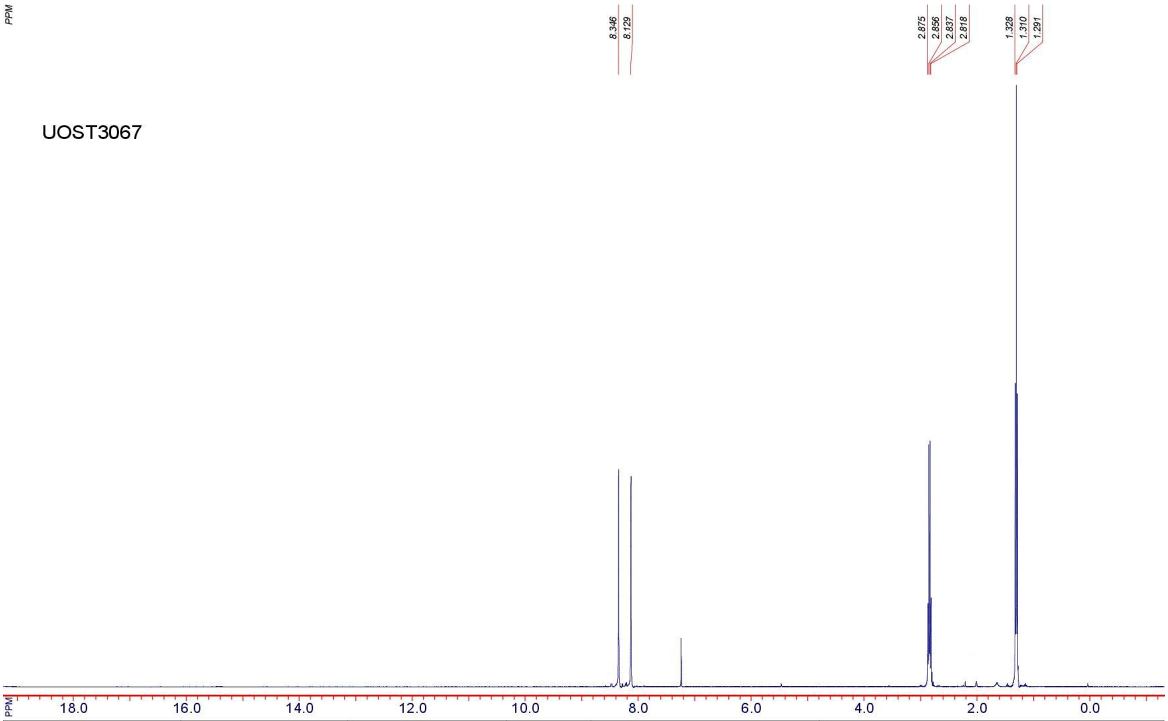

**File name: UOST3067**	Operator: USER 1	SF: 400.1300 MHz	NSC: 1	PW: 0.00 usec, RG: 25	SI: 32768
Date: 06-Mar-2017	Solvent: CDCl3	SW: 8224 Hz	TE: 297 K	AQ: 1.99 sec, RD: 0.00 sec	Parameter file, TOPSPIN Version 2.1

*9.2. 2-Ethyl-5-(trifluoromethoxy)pyrazine **11** (^{13}C NMR).*

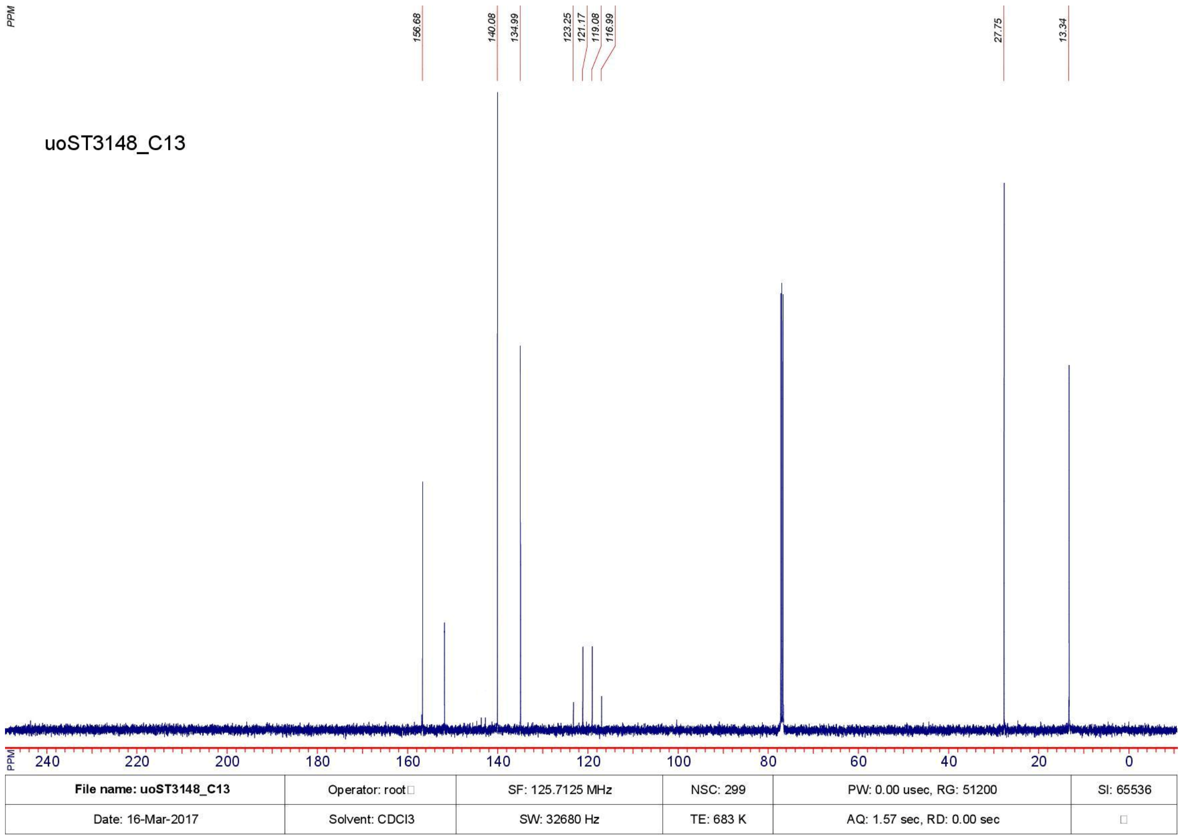

*9.3. 2-Ethyl-5-(trifluoromethoxy)pyrazine **11** (^{19}F NMR).*

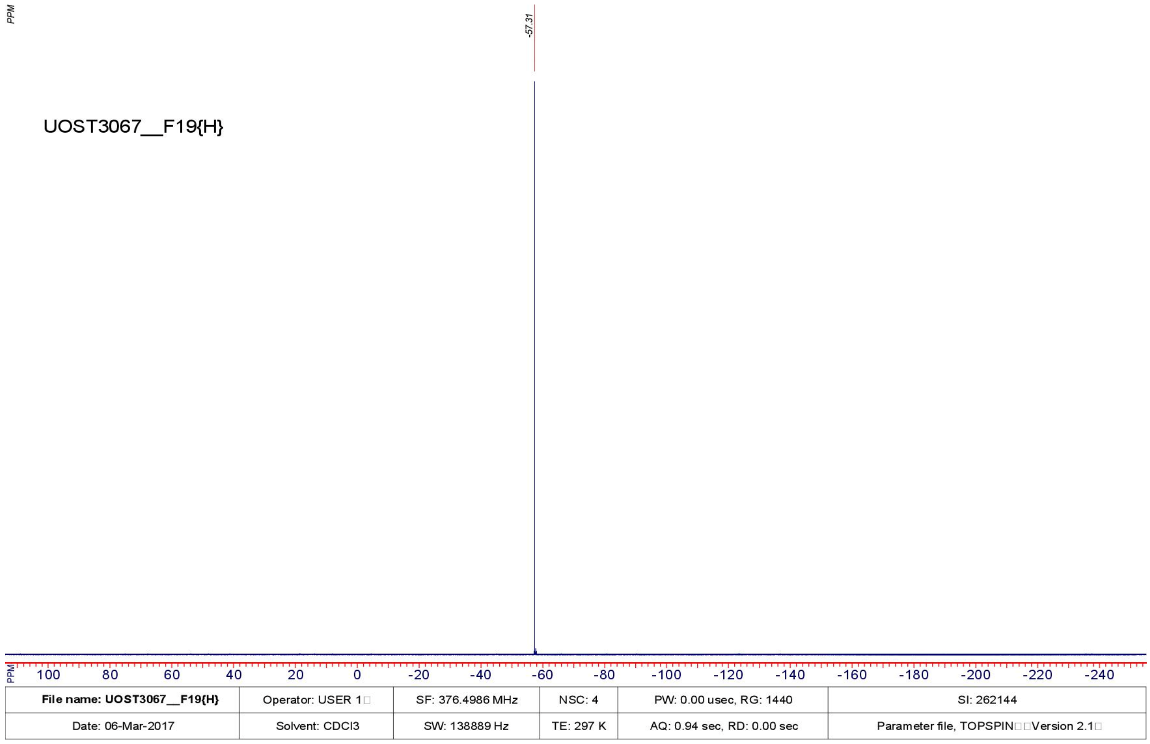

10.1. 2-Butyl-5-(trifluoromethoxy)pyrazine ***12*** *(^{1}H NMR).*

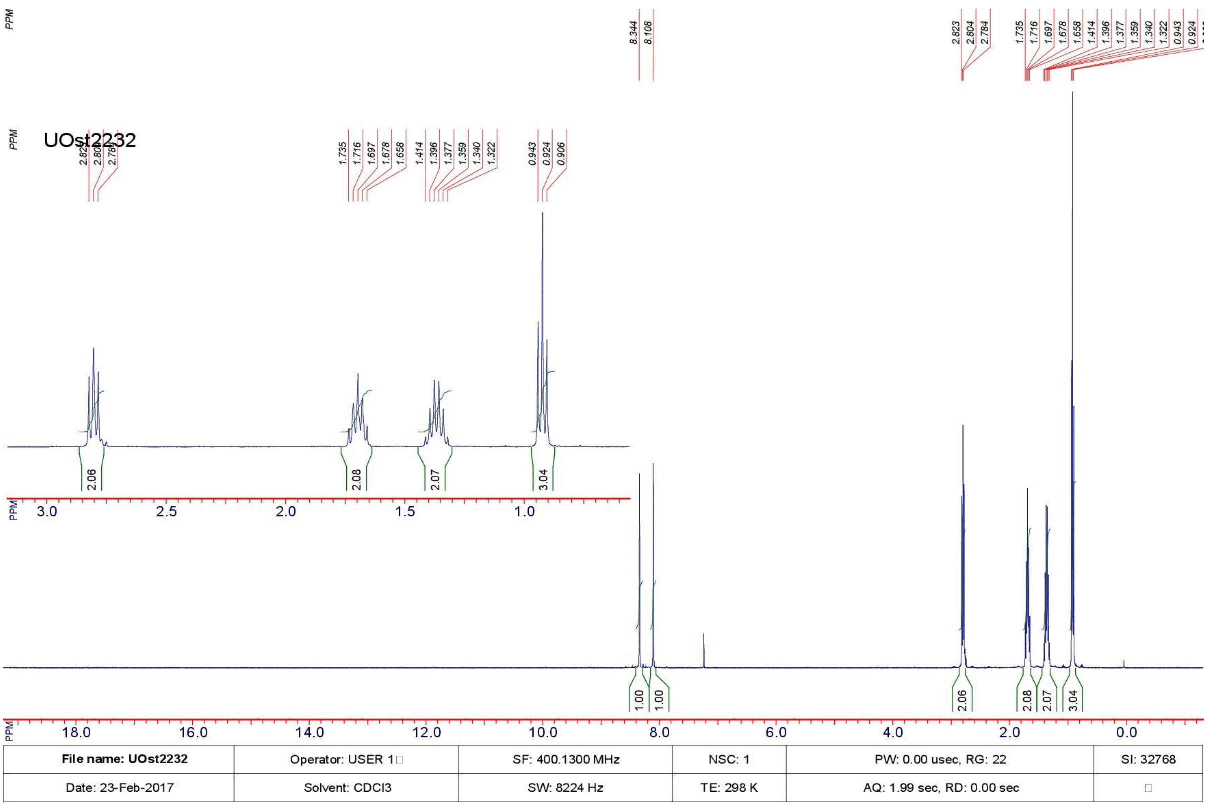

10.2. 2-Butyl-5-(trifluoromethoxy)pyrazine ***12*** *(^{13}C NMR).*

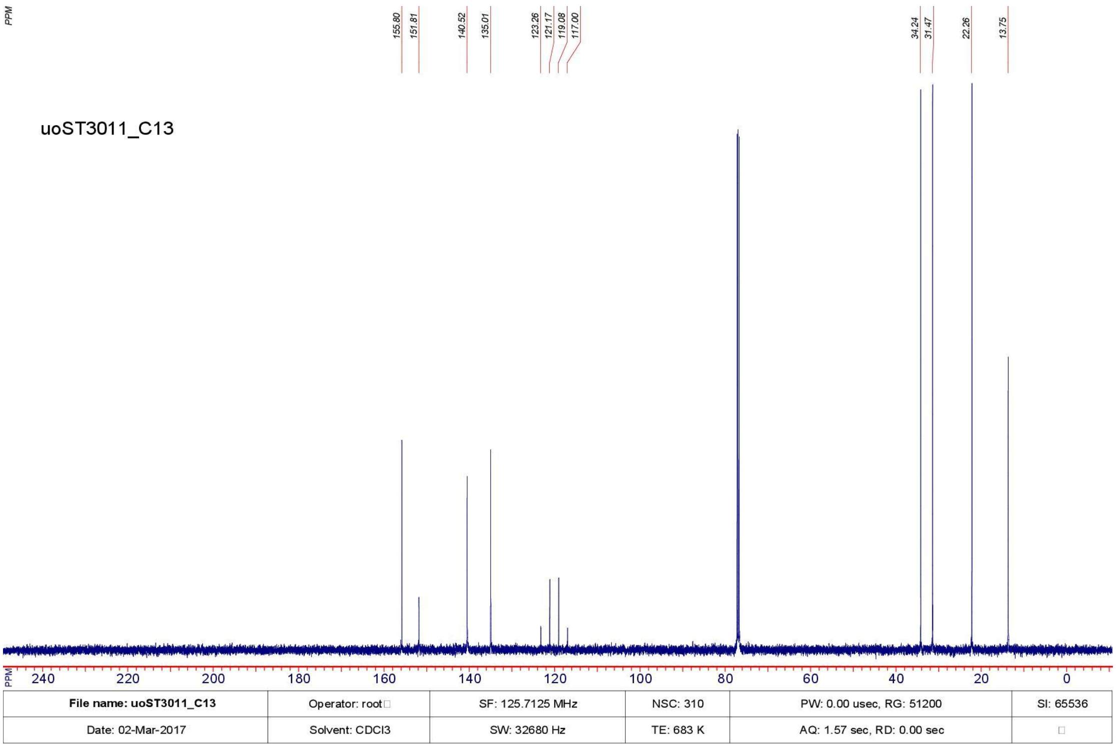

*10.3. 2-Butyl-5-(trifluoromethoxy)pyrazine **12** (^{19}F NMR).*

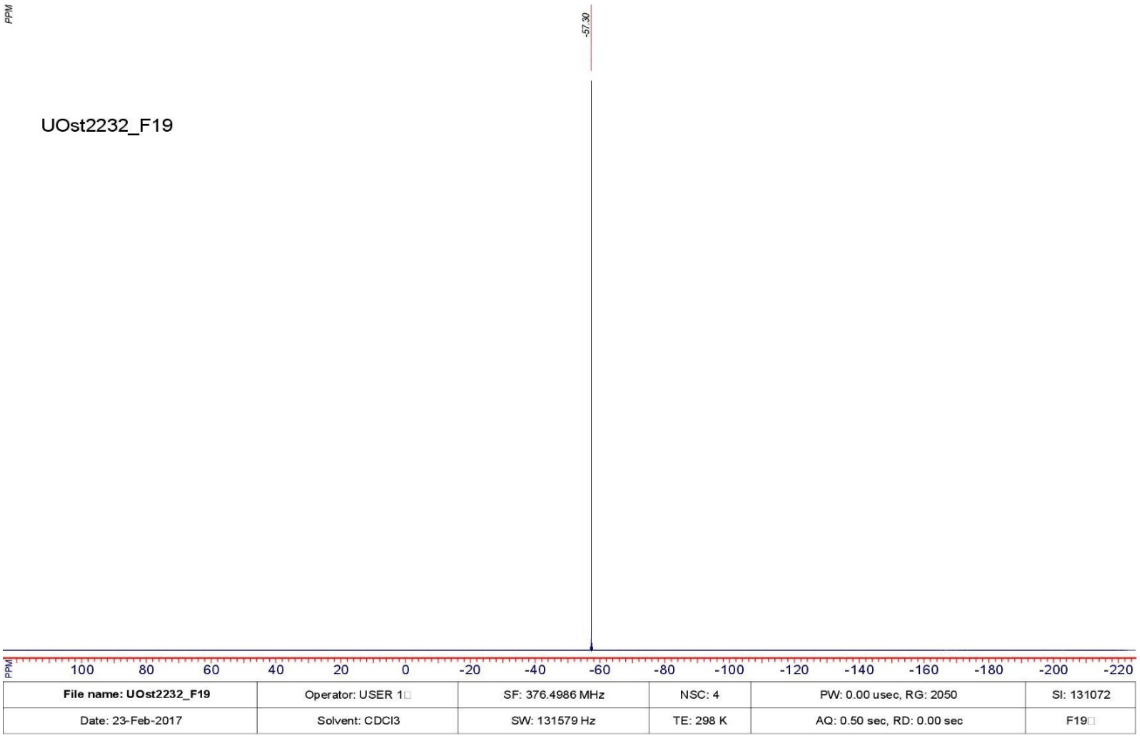

*11.1. 2-Phenyl-5-(trifluoromethoxy)pyrazine **13** (^{1}H NMR).*

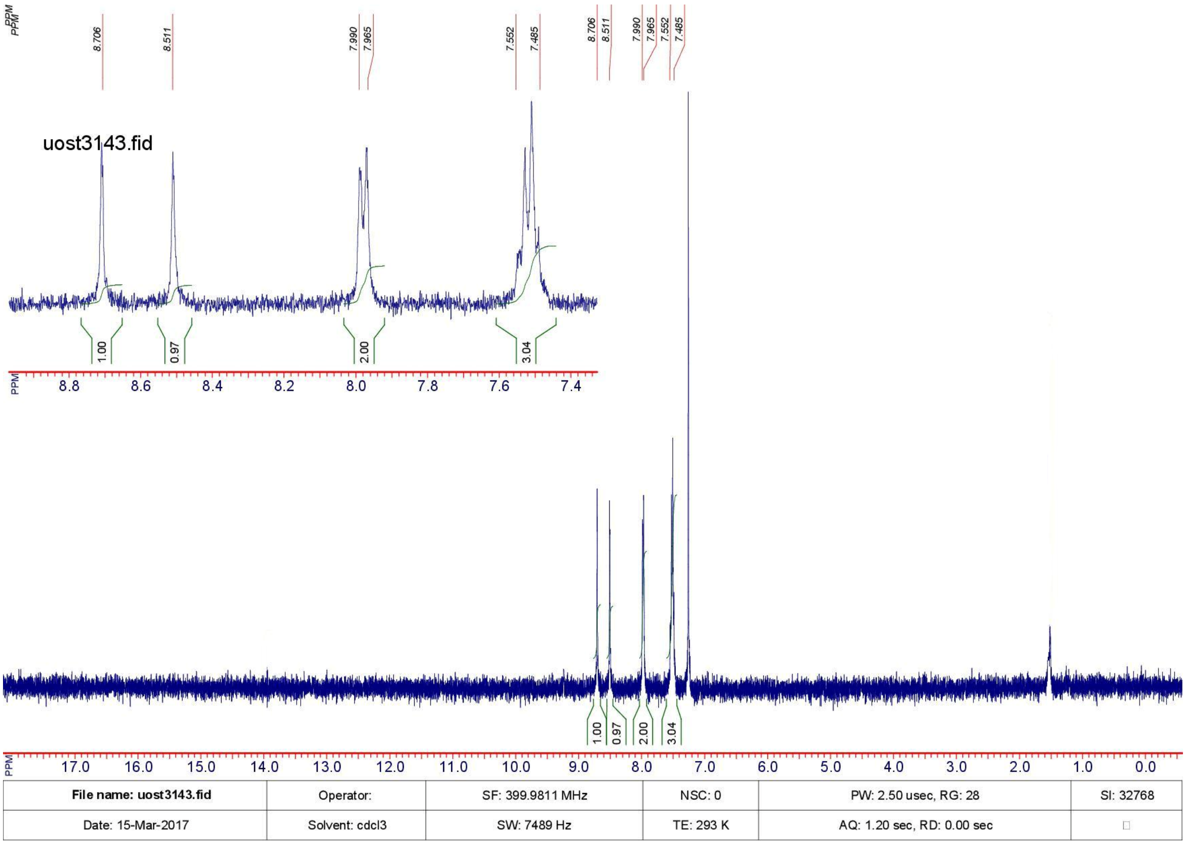

*11.2. 2-Phenyl-5-(trifluoromethoxy)pyrazine **13** (^{13}C NMR).*

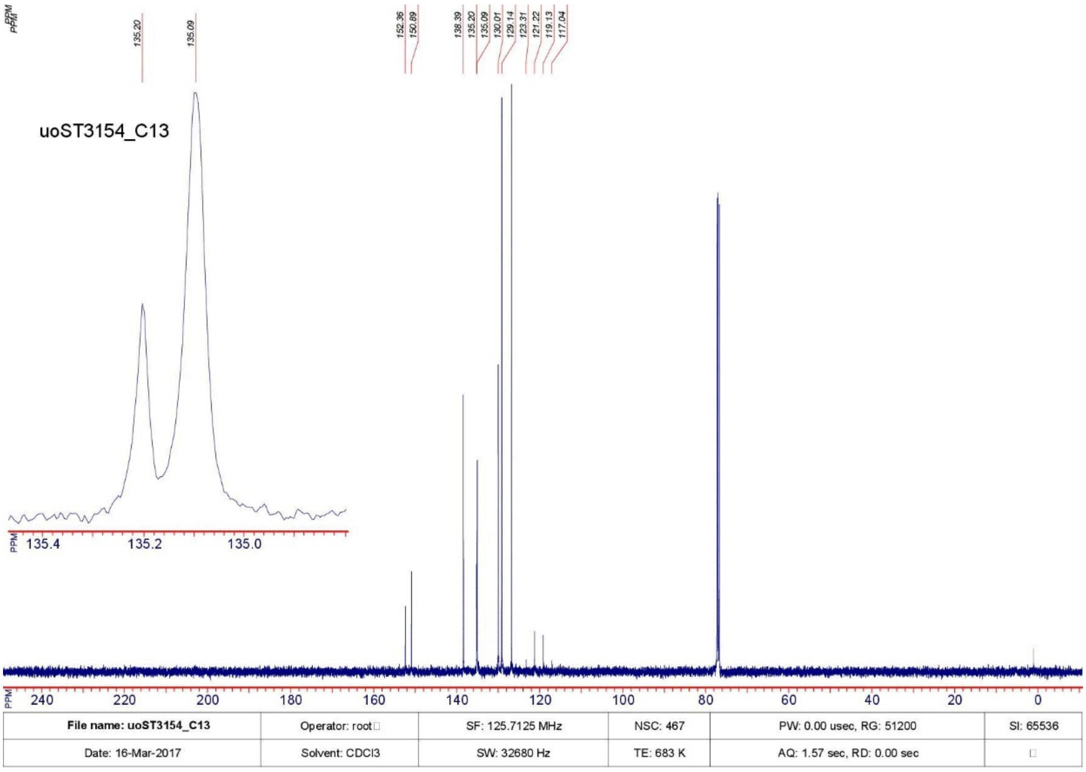

*11.3. 2-Phenyl-5-(trifluoromethoxy)pyrazine **13** (^{19}F NMR).*

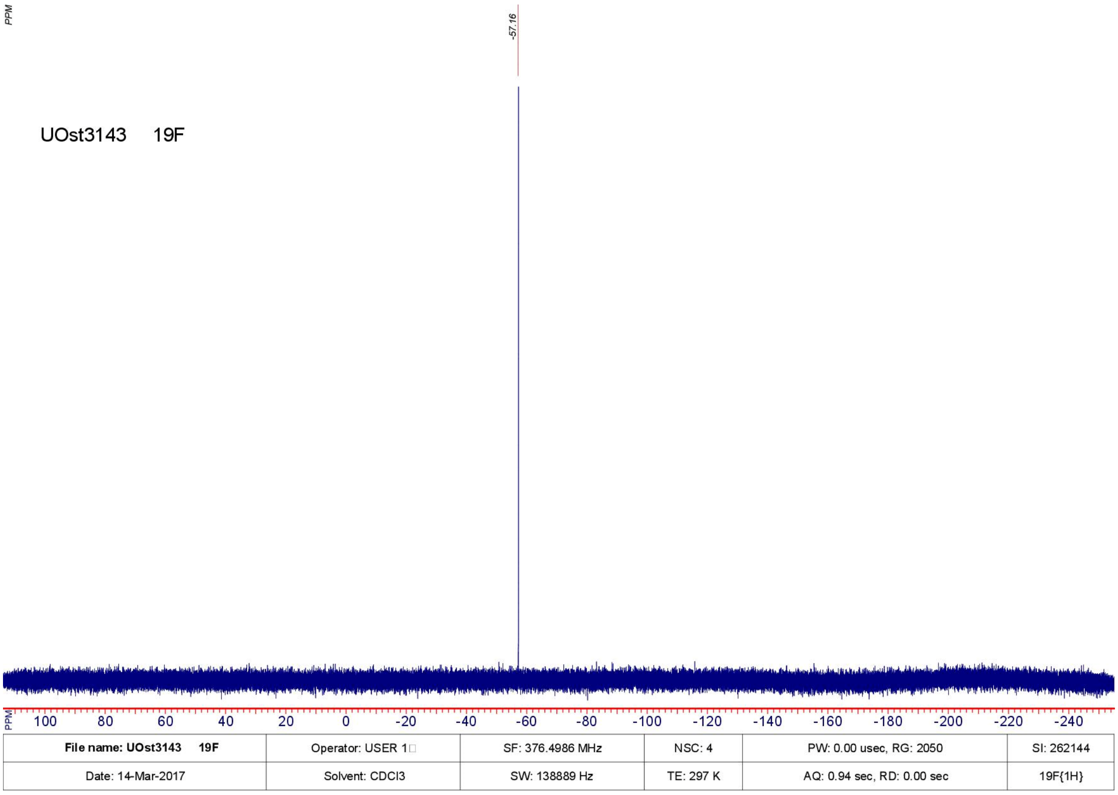

12.1. 2-(Trifluoromethoxy)-5-vinylpyrazine **14** (^{1}H NMR).

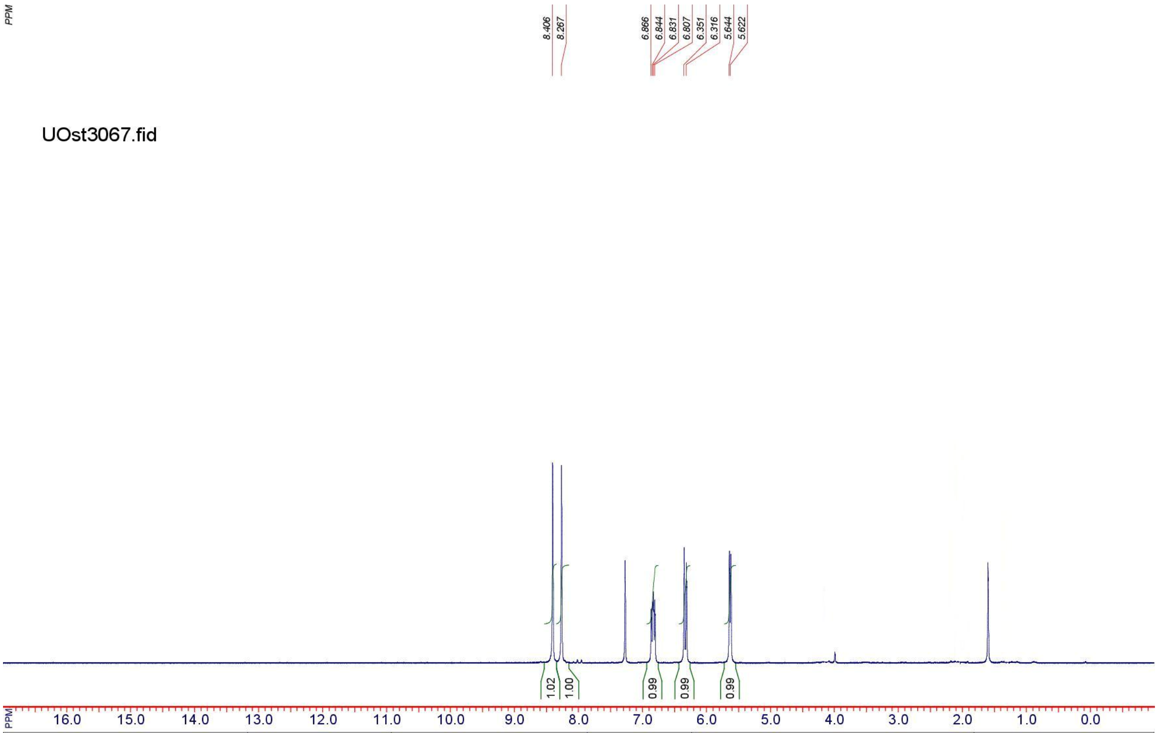

**File name: UOst3067.fid**	Operator:	SF: 499.8255 MHz	NSC: 0	PW: 9.80 usec, RG: 12	SI: 32768
Date: 06-Mar-2019	Solvent: cdcl3	SW: 8993 Hz	TE: 323 K	AQ: 1.78 sec, RD: 0.00 sec	new experiment□

12.2. 2-(Trifluoromethoxy)-5-vinylpyrazine **14** (^{13}C NMR).

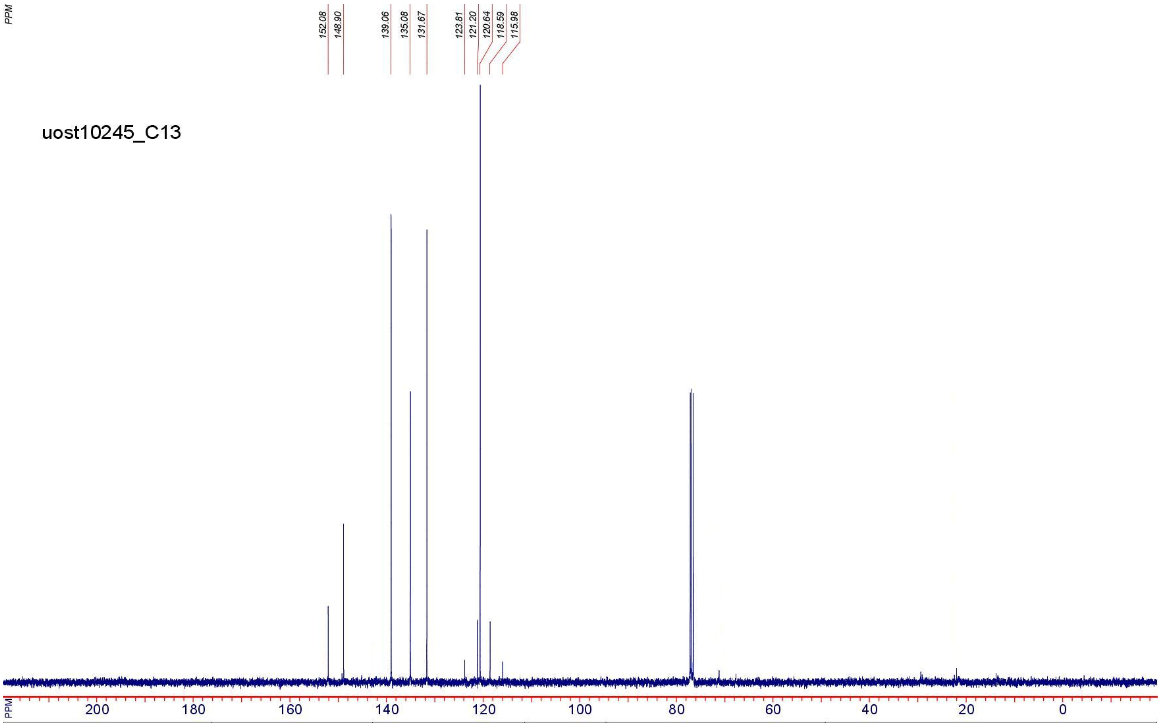

**File name: uost10245_C13**	Operator: USER 1□	SF: 100.6128 MHz	NSC: 264	PW: 0.00 usec, RG: 1820	SI: 65536
Date: 24-Oct-2018	Solvent: DMSO	SW: 24038 Hz	TE: 299 K	AQ: 1.36 sec, RD: 0.00 sec	Parameter file, TOPSPIN□□Version 2.1□

*12.3. 2-(Trifluoromethoxy)-5-vinylpyrazine **14** (^{19}F NMR).*

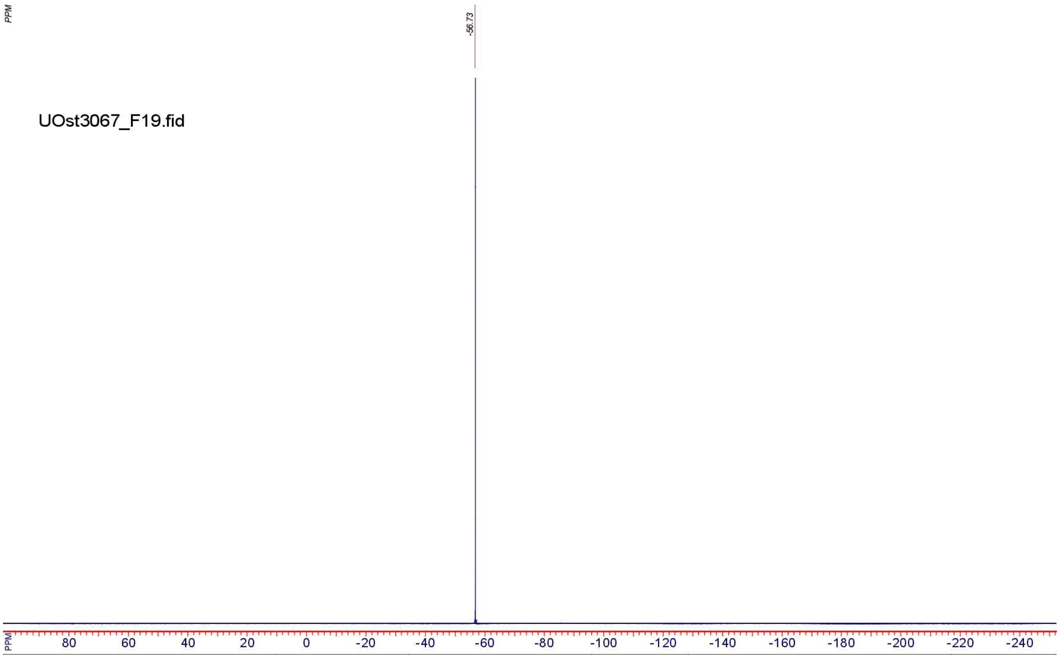

**File name: UOst3067_F19.fid**	Operator:	SF: 470.2668 MHz	NSC: 0	PW: 6.67 usec, RG: 12	SI: 524288
Date: 06-Mar-2019	Solvent: cdcl3	SW: 166667 Hz	TE: 323 K	AQ: 1.36 sec, RD: 0.00 sec	

*13.1. 2,5-Divinylpyrazine **15** (^{1}H NMR).*

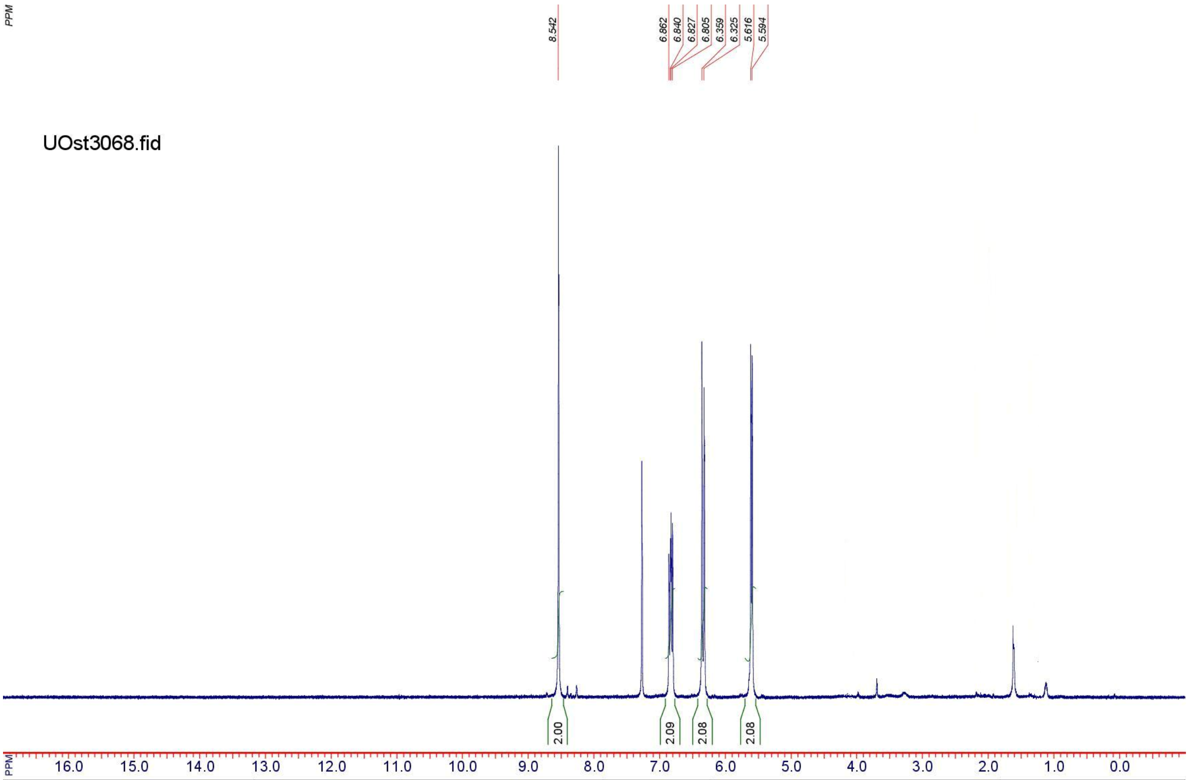

**File name: UOst3068.fid**	Operator:	SF: 499.8255 MHz	NSC: 0	PW: 9.80 usec, RG: 12	SI: 32768
Date: 06-Mar-2019	Solvent: cdcl3	SW: 8993 Hz	TE: 323 K	AQ: 1.78 sec, RD: 0.00 sec	new experiment

*13.2. 2,5-Divinylpyrazine **15** (^{13}C NMR).*

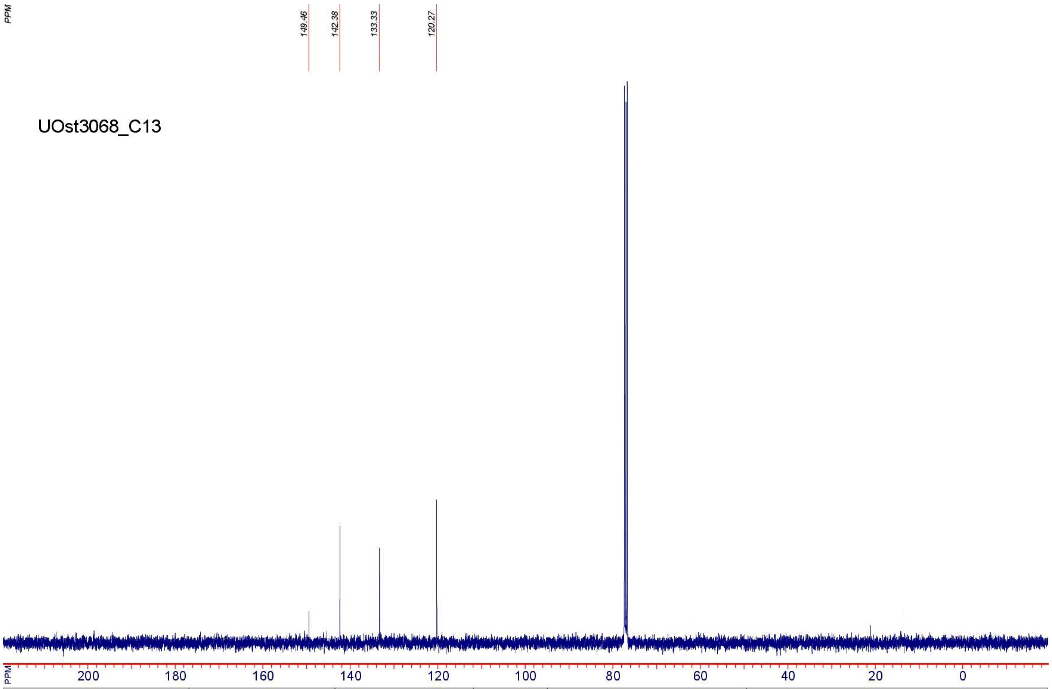

File name: UOst3068_C13	Operator: USER 1	SF: 100.6128 MHz	NSC: 658	PW: 0.00 usec, RG: 2050	SI: 32768
Date: 11-Mar-2019	Solvent: CDCl3	SW: 24038 Hz	TE: 296 K	AQ: 1.06 sec, RD: 0.00 sec	Parameter file, TOPSPIN Version 2.1

*14.1. 2-(Trifluoromethoxy)-5-((trimethylsilyl)ethynyl)pyrazine **16** (^{1}H NMR).*

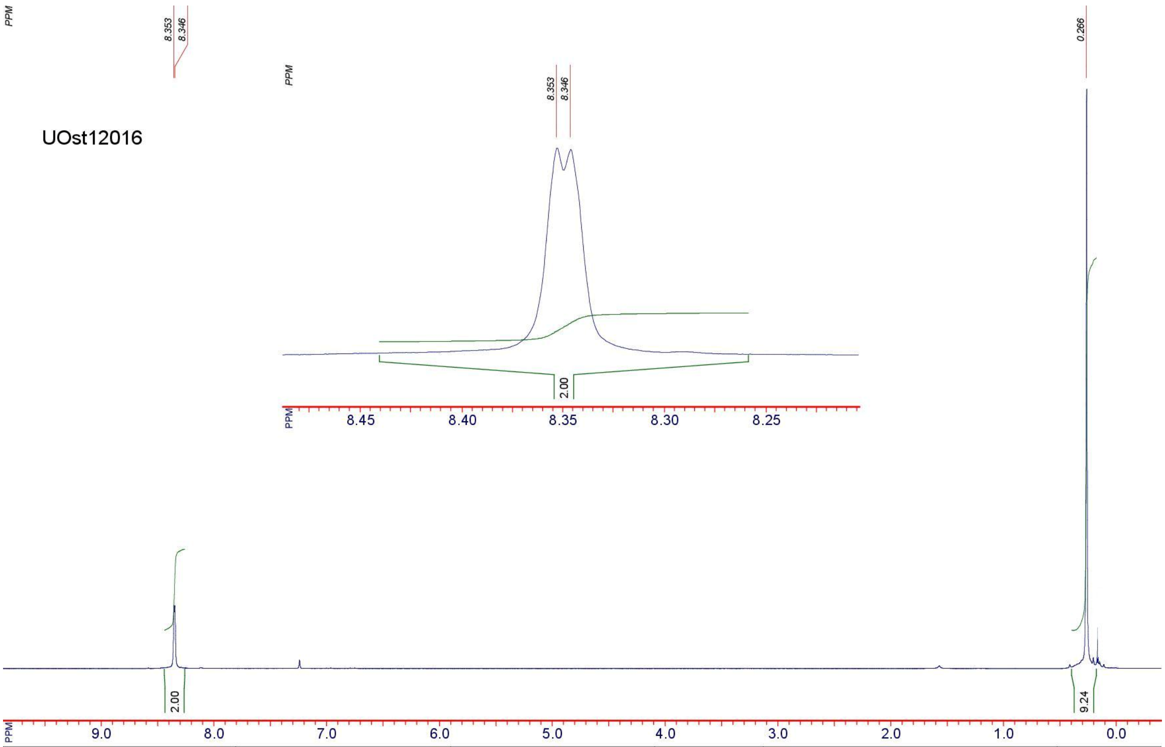

File name: UOst12016	Operator: USER 1	SF: 400.1300 MHz	NSC: 1	PW: 0.00 usec, RG: 25	SI: 32768
Date: 01-Dec-2016	Solvent: CDCl3	SW: 8224 Hz	TE: 297 K	AQ: 1.99 sec, RD: 0.00 sec	

14.2. *2-(Trifluoromethoxy)-5-((trimethylsilyl)ethynyl)pyrazine* ***16*** *(^{13}C NMR).*

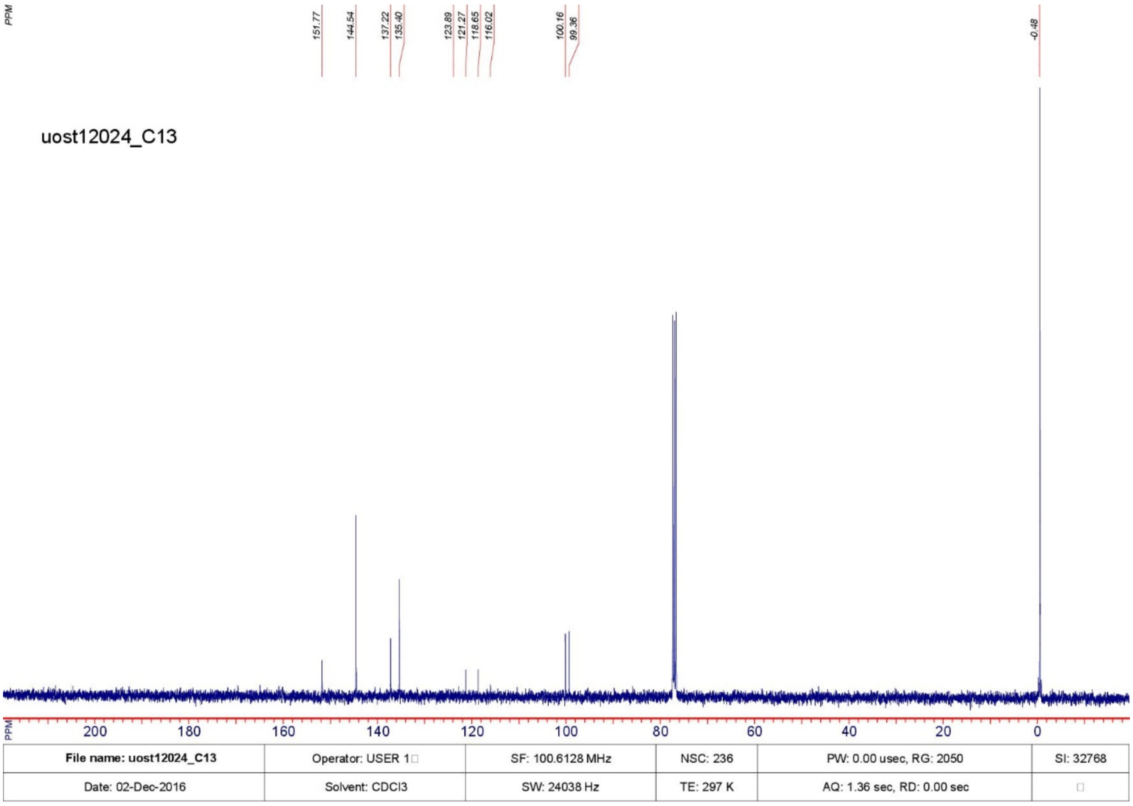

14.3. *2-(Trifluoromethoxy)-5-((trimethylsilyl)ethynyl)pyrazine* ***16*** *(^{19}F NMR).*

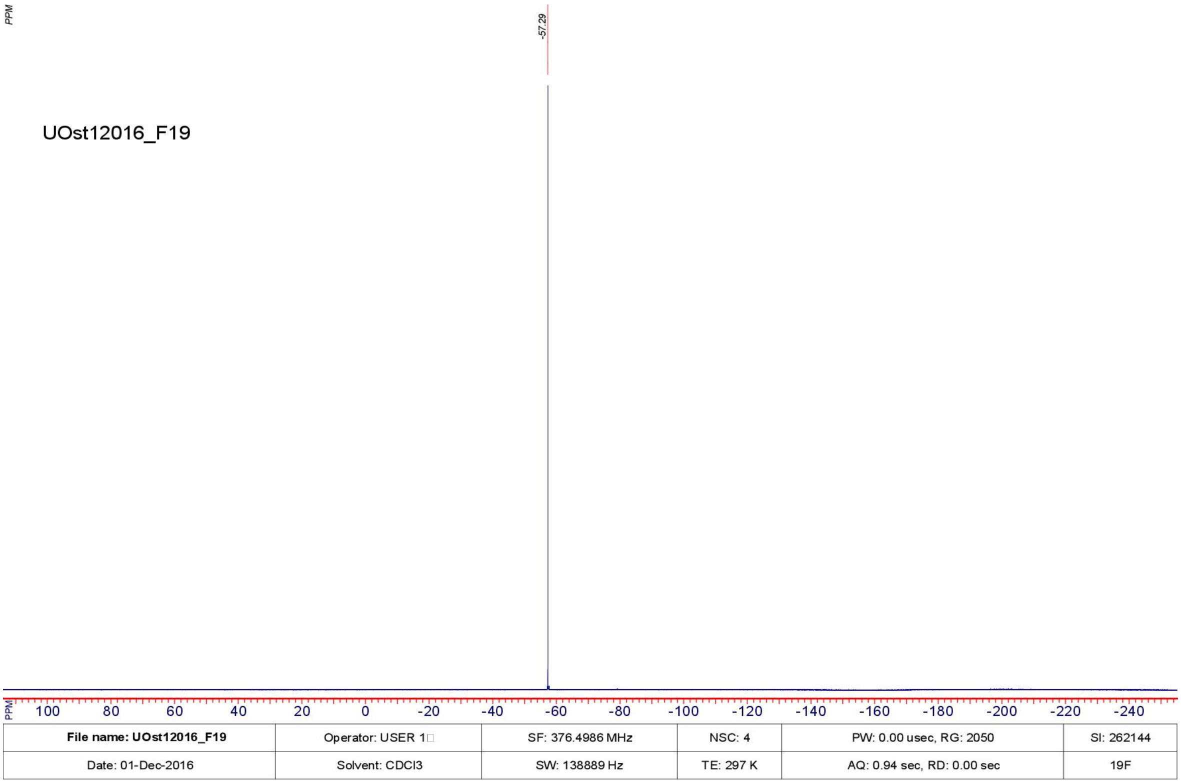

*15.1. 2-Ethynyl-5-(trifluoromethoxy)pyrazine **17** (^{1}H NMR).*

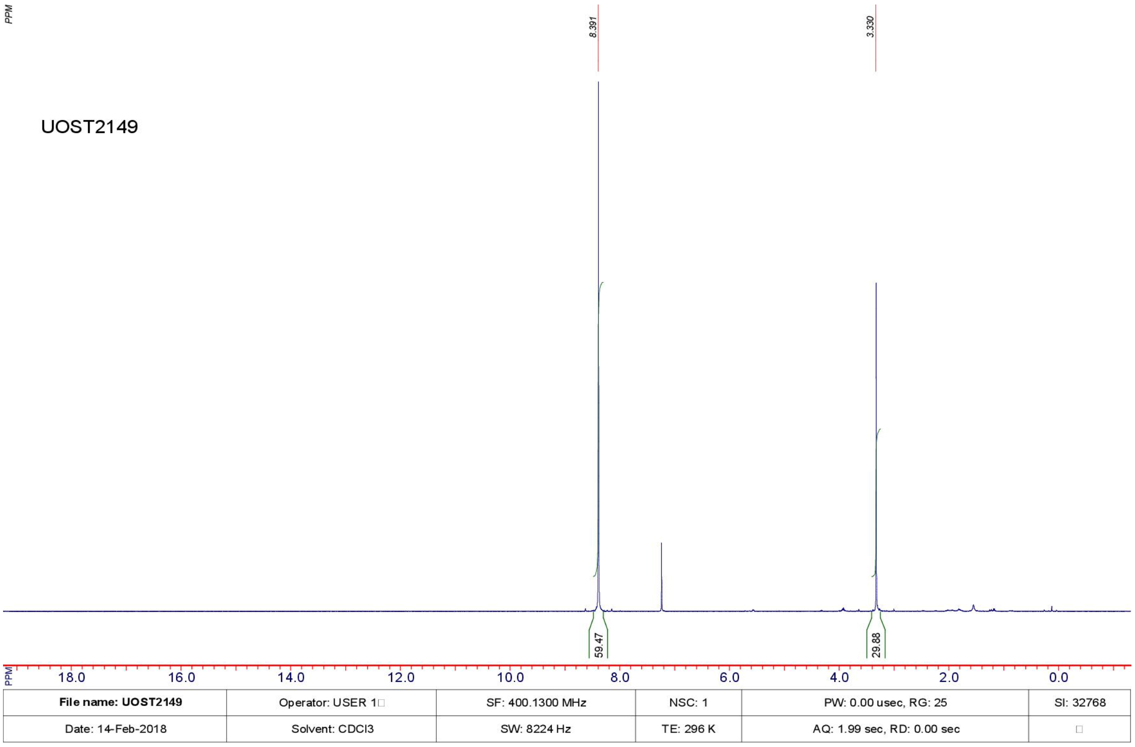

*15.2. 2-Ethynyl-5-(trifluoromethoxy)pyrazine **17** (^{13}C NMR).*

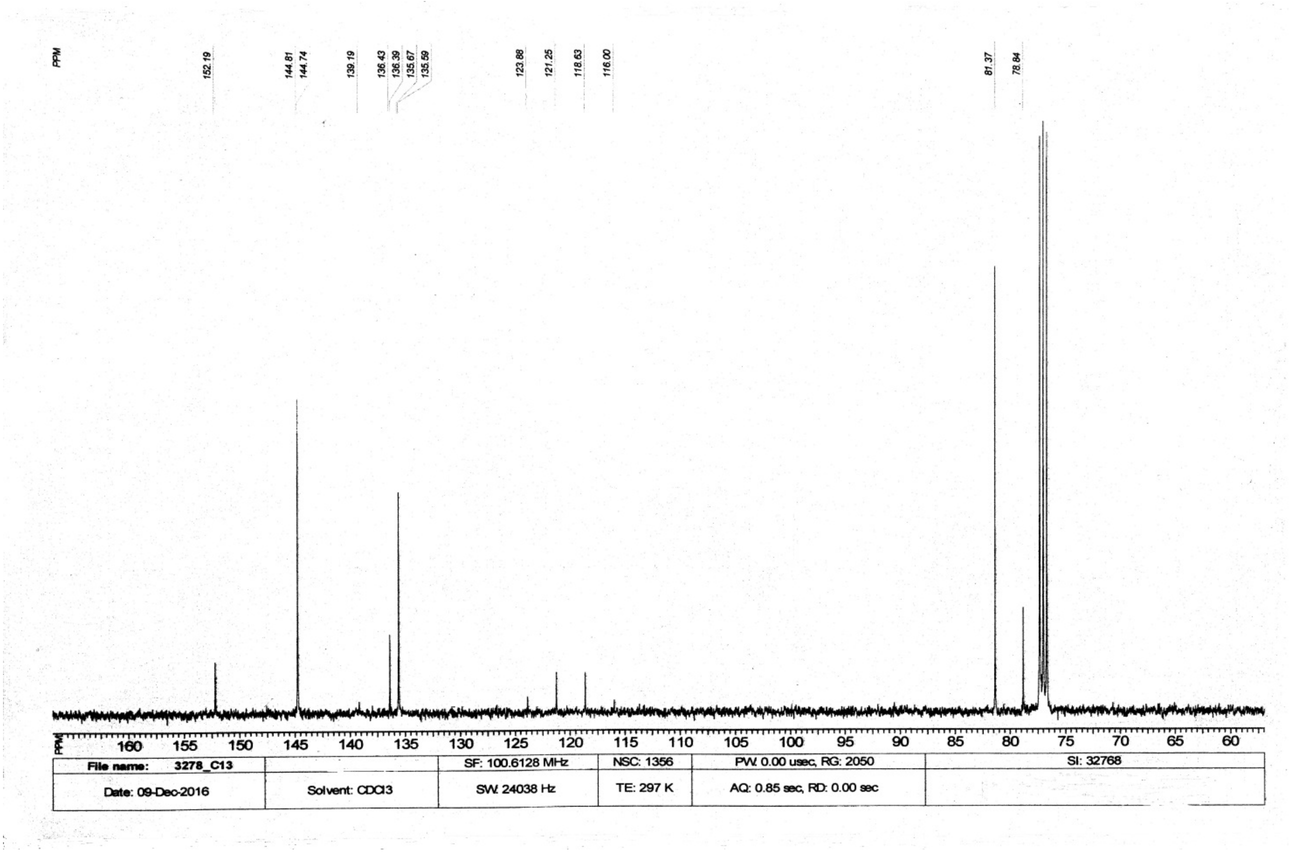

*15.3. 2-Ethynyl-5-(trifluoromethoxy)pyrazine **17** (^{19}F NMR).*

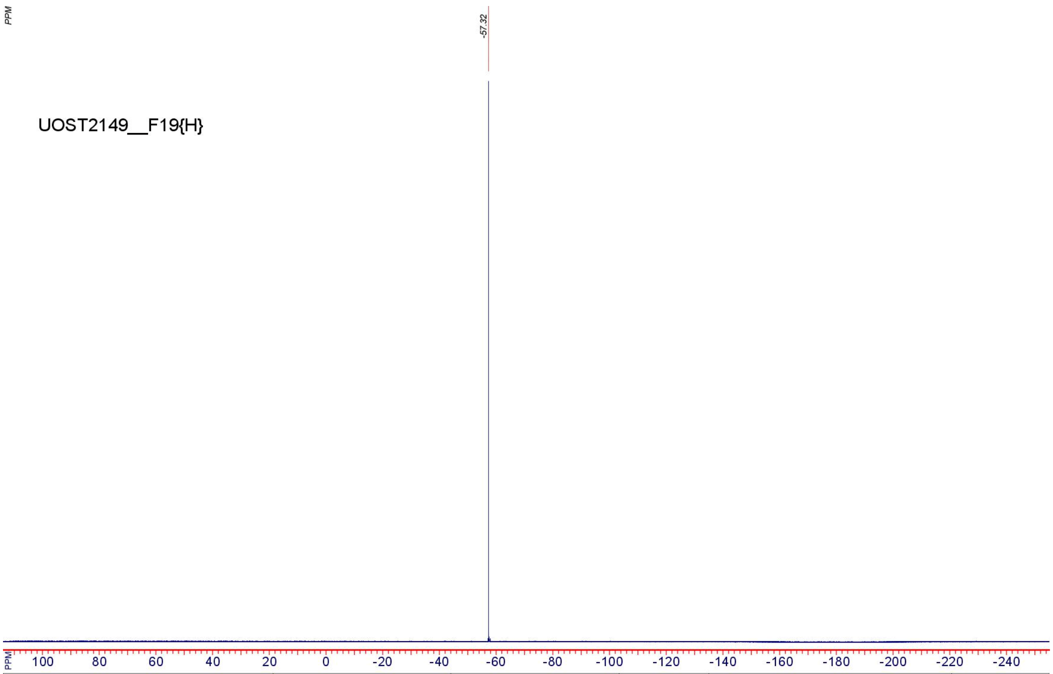

File name: UOST2149__F19{H}	Operator: USER 1	SF: 376.4986 MHz	NSC: 2	PW: 0.00 usec, RG: 2050	SI: 262144
Date: 14-Feb-2018	Solvent: CDCl3	SW: 138889 Hz	TE: 296 K	AQ: 0.94 sec, RD: 0.00 sec	

*16.1. 2-Ethynyl-5-methoxypyrazine **18** (^{1}H NMR).*

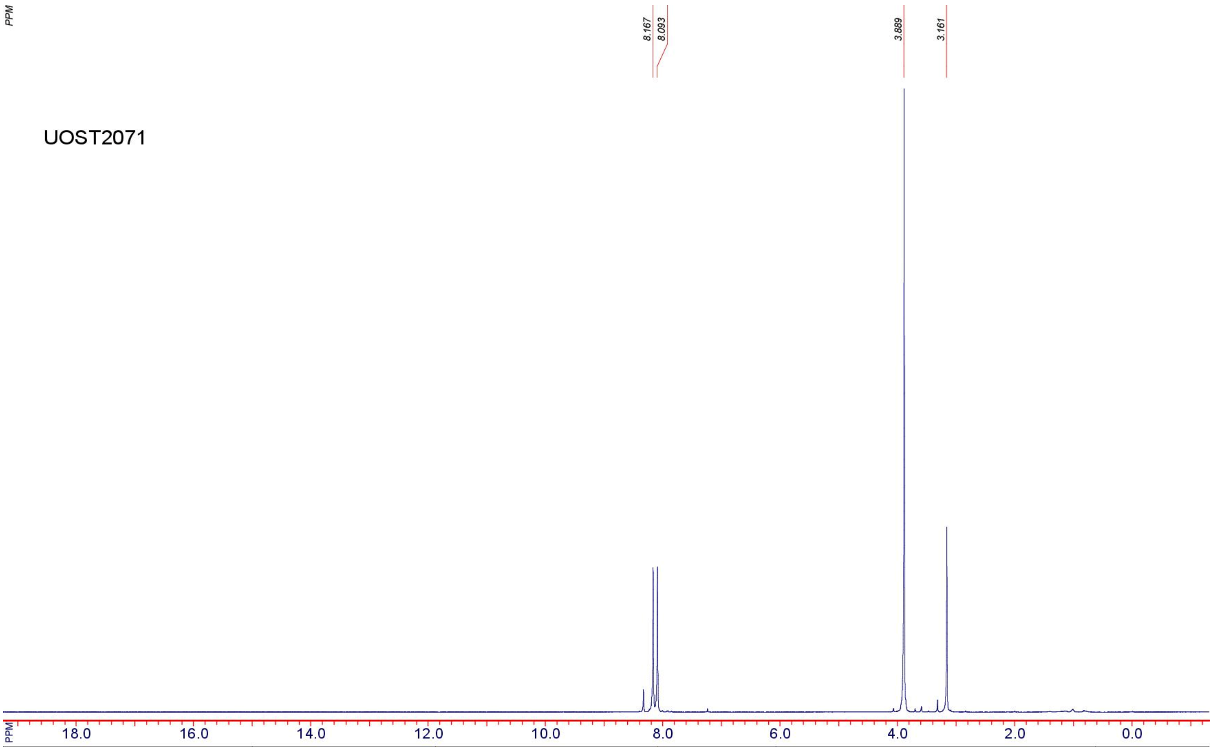

File name: UOST2071	Operator: nmr	SF: 400.1300 MHz	NSC: 1	PW: 0.00 usec, RG: 25	SI: 32768
Date: 07-Feb-2020	Solvent: CDCl3	SW: 8224 Hz	TE: 296 K	AQ: 1.99 sec, RD: 0.00 sec	

*16.2. 2-Ethynyl-5-methoxypyrazine **18** (^{13}C NMR).*

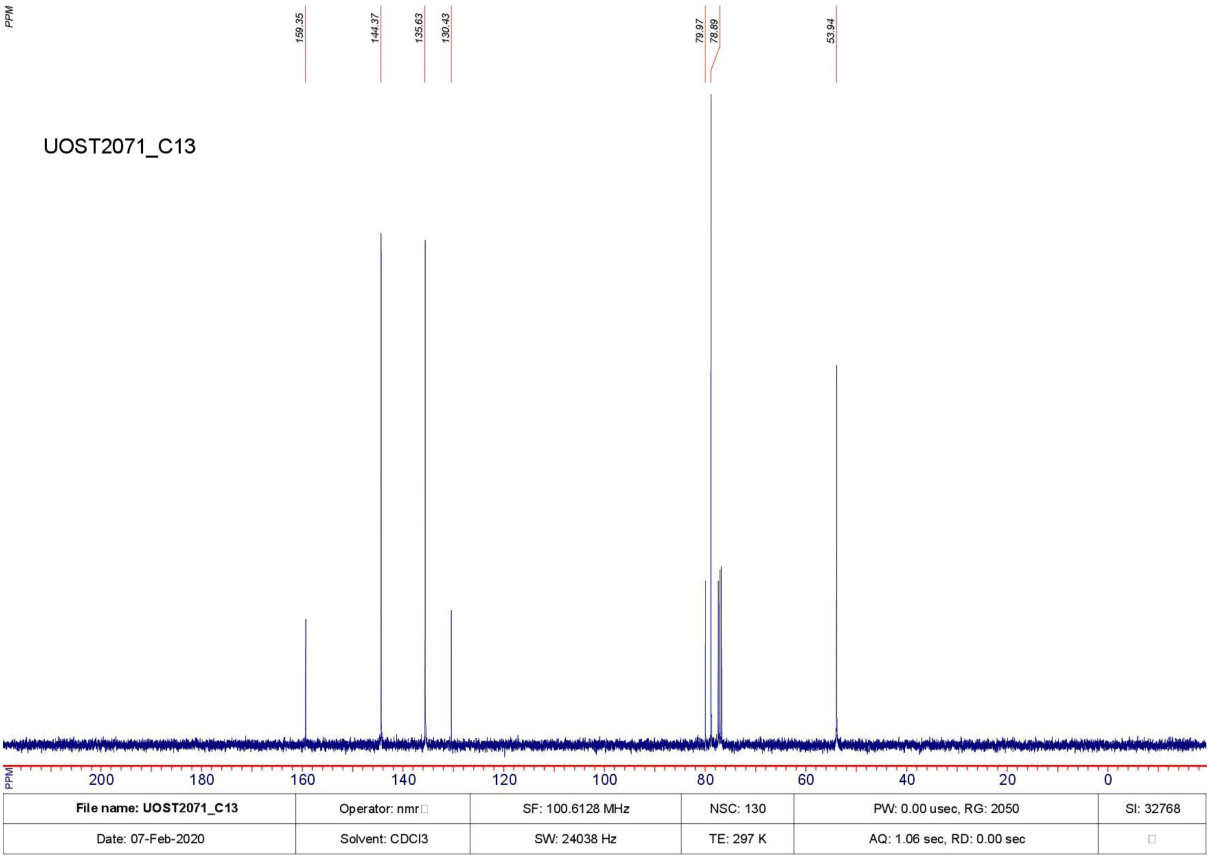

*17.1. 1-(5-(trifluoromethoxy)pyrazin-2-yl)ethan-1-one **19** (^{1}H NMR).*

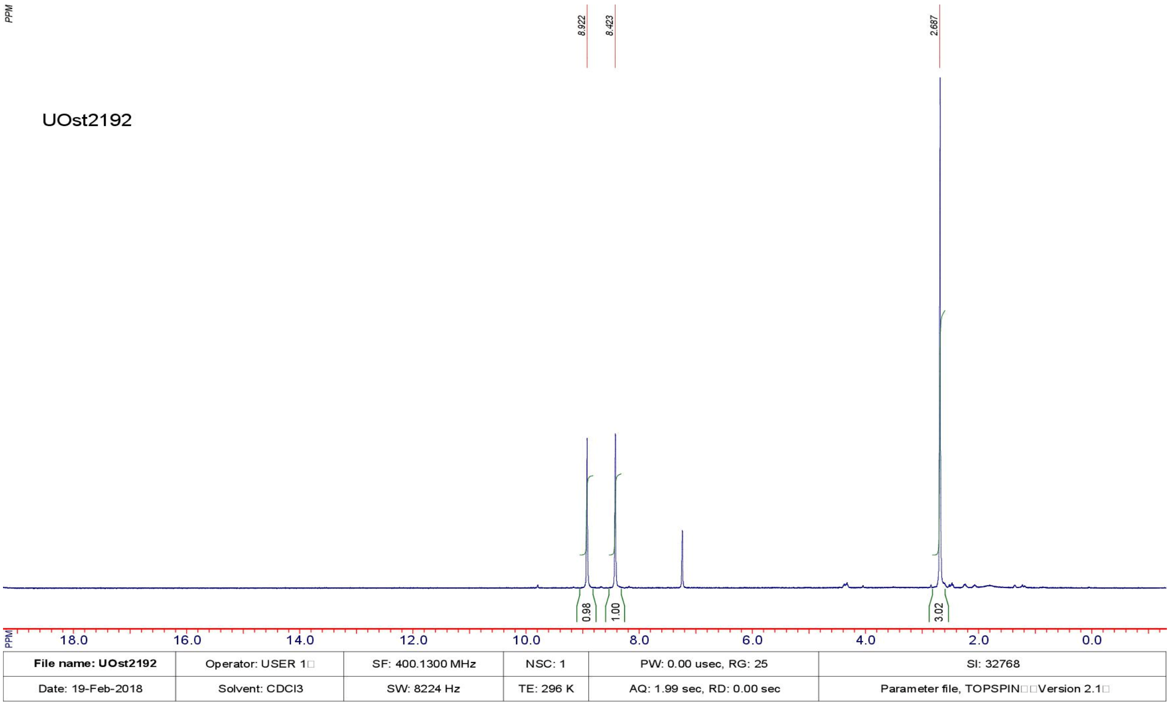

*17.2. 1-(5-(trifluoromethoxy)pyrazin-2-yl)ethan-1-one **19** (^{13}C NMR).*

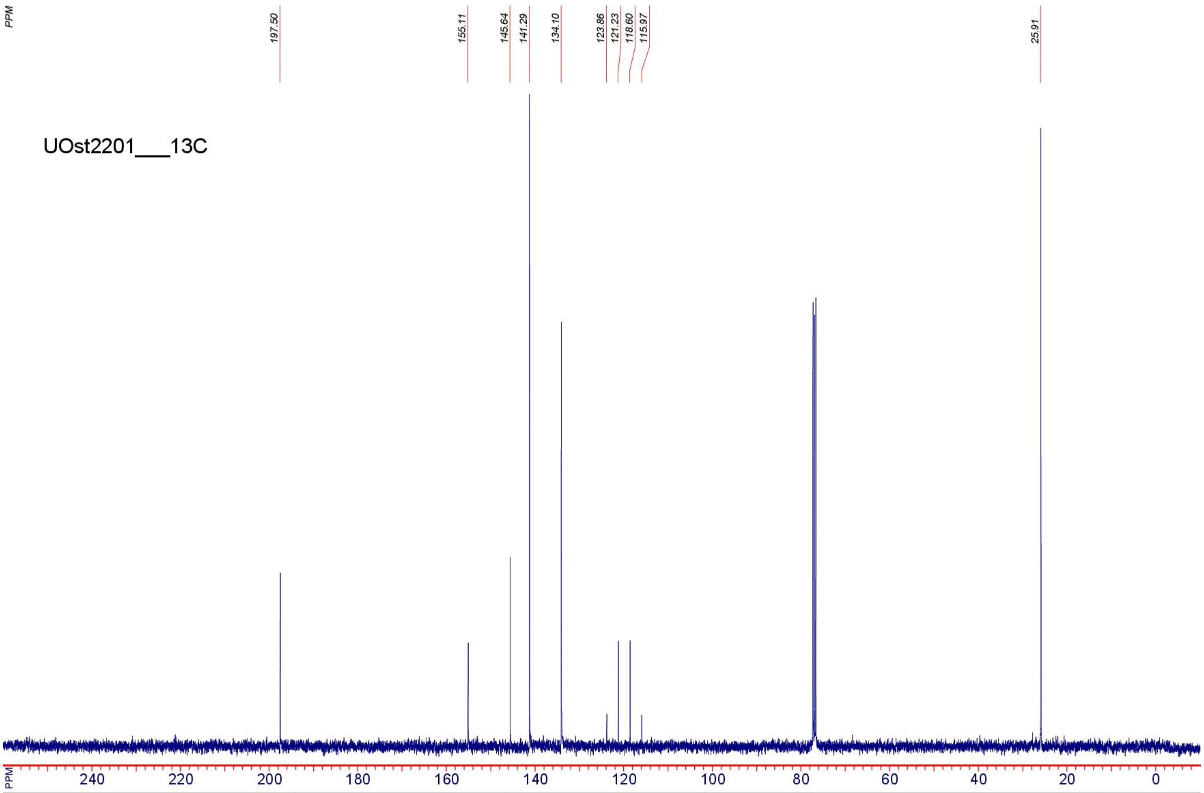

File name: UOst2201___13C	Operator: USER 1	SF: 100.6128 MHz	NSC: 416	PW: 0.00 usec, RG: 2050	SI: 32768
Date: 20-Feb-2018	Solvent: CDCl3	SW: 27174 Hz	TE: 296 K	AQ: 0.94 sec, RD: 0.00 sec	

*17.3. 1-(5-(trifluoromethoxy)pyrazin-2-yl)ethan-1-one **19** (^{19}F NMR).*

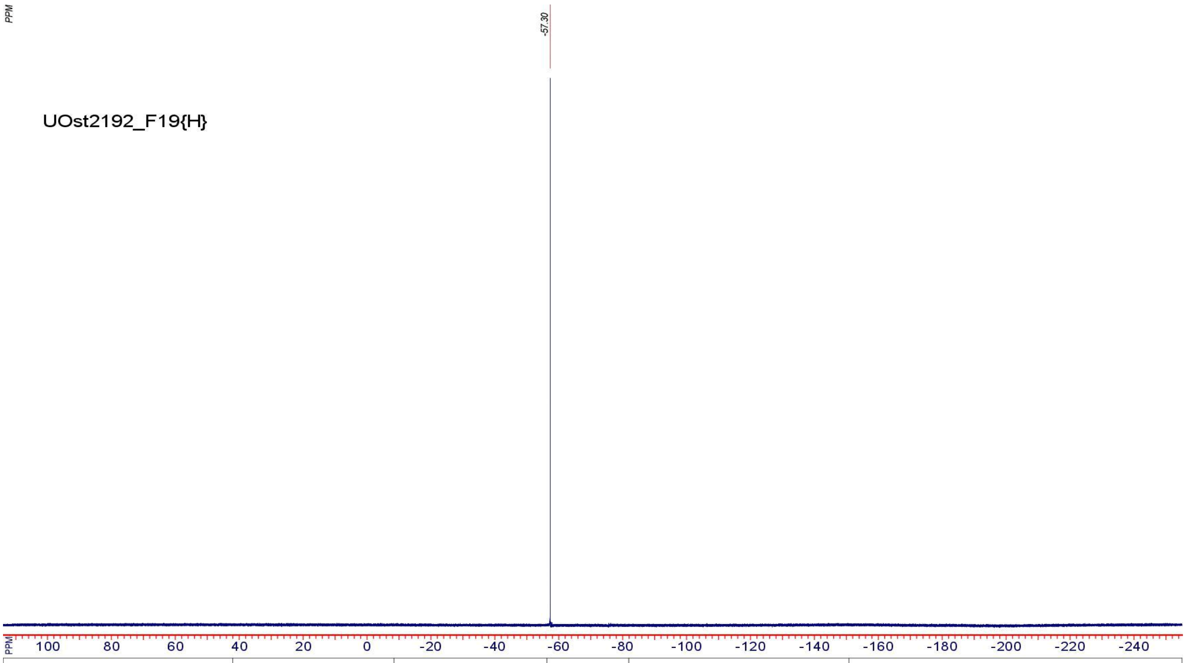

File name: UOst2192_F19{H}	Operator: USER 1	SF: 376.4986 MHz	NSC: 2	PW: 0.00 usec, RG: 2050	SI: 262144
Date: 19-Feb-2018	Solvent: CDCl3	SW: 138889 Hz	TE: 296 K	AQ: 0.94 sec, RD: 0.00 sec	Parameter file, TOPSPIN Version 2.1
